# Monoclonal antibodies for COVID-19 therapy and SARS-CoV-2 detection

**DOI:** 10.1186/s12929-021-00784-w

**Published:** 2022-01-04

**Authors:** Yu-Chyi Hwang, Ruei-Min Lu, Shih-Chieh Su, Pao-Yin Chiang, Shih-Han Ko, Feng-Yi Ke, Kang-Hao Liang, Tzung-Yang Hsieh, Han-Chung Wu

**Affiliations:** 1 Institute of Cellular and Organismic Biology, Academia Sinica, No. 128, Academia Road, Section 2, Nankang, Taipei, 11529 Taiwan; 2 Biomedical Translation Research Center (BioTReC), Academia Sinica, Taipei, 11529 Taiwan

**Keywords:** Angiotensin converting enzyme II (ACE2), Coronavirus disease 2019 (COVID-19), Cytokine storm, Viral detection, Severe acute respiratory syndrome coronavirus 2 (SARS-CoV-2), Spike, Receptor-binding domain (RBD), Receptor binding motif (RBM), Therapeutic antibody

## Abstract

The coronavirus disease 2019 (COVID-19) pandemic is an exceptional public health crisis that demands the timely creation of new therapeutics and viral detection. Owing to their high specificity and reliability, monoclonal antibodies (mAbs) have emerged as powerful tools to treat and detect numerous diseases. Hence, many researchers have begun to urgently develop Ab-based kits for the detection of severe acute respiratory syndrome coronavirus 2 (SARS-CoV-2) and Ab drugs for use as COVID-19 therapeutic agents. The detailed structure of the SARS-CoV-2 spike protein is known, and since this protein is key for viral infection, its receptor-binding domain (RBD) has become a major target for therapeutic Ab development. Because SARS-CoV-2 is an RNA virus with a high mutation rate, especially under the selective pressure of aggressively deployed prophylactic vaccines and neutralizing Abs, the use of Ab cocktails is expected to be an important strategy for effective COVID-19 treatment. Moreover, SARS-CoV-2 infection may stimulate an overactive immune response, resulting in a cytokine storm that drives severe disease progression. Abs to combat cytokine storms have also been under intense development as treatments for COVID-19. In addition to their use as drugs, Abs are currently being utilized in SARS-CoV-2 detection tests, including antigen and immunoglobulin tests. Such Ab-based detection tests are crucial surveillance tools that can be used to prevent the spread of COVID-19. Herein, we highlight some key points regarding mAb-based detection tests and treatments for the COVID-19 pandemic.

## Introduction

The coronavirus disease 2019 (COVID-19) pandemic is the result of widespread infection with severe acute respiratory syndrome coronavirus 2 (SARS-CoV-2). Compared to other highly transmissible viruses, SARS-CoV-2 is associated with high rates of morbidity and mortality, and it represents an unprecedented challenge to global public health [1]. Most people infected with SARS-CoV-2 experience mild to moderate respiratory illness similar to influenza or other virus infections, with symptoms such as fever, dry cough, and dyspnea. However, a considerable number of infected people develop pneumonia and acute lung injury or acute respiratory distress syndrome (ARDS); these conditions are closely associated with the relatively high mortality rate of COVID-19 [2]. Some patients also exhibit pulmonary alveolitis, bronchiolitis, accumulation of mucus and edema fluid, and different degrees of inflammation marked by infiltration of various immune cells into the pulmonary interstitium [3, 4].

The tissue distribution of the virus-targeted receptor protein, angiotensin converting enzyme II (ACE2), determines which organs will be attacked by SARS‐CoV‐2; lung, the immune system, heart, kidney, esophagus and small intestine all have high expression of ACE2 [5–8]. Based on this set of target tissues, SARS-CoV-2 can cause non‐respiratory clinical symptoms, such as diarrhea, sore throat, muscle aches, headache and vomiting, in a minority of patients [8, 9]. Moreover, patients with severe disease suffer from respiratory and lung function failure, and some even require extracorporeal membrane oxygenation (ECMO) and intensive care due to multiple organ failure and septic shock [6, 10]. Therefore, a pressing global need exists to develop vaccines and therapeutics that can mitigate the COVID-19 pandemic and cure infected patients.

Over the past year, extraordinary biomedical and financial resources have been devoted to the rapid development of diagnostic, prophylactic and therapeutic measures for this single disease. Due to their high specificity and versatility, monoclonal antibodies (mAbs) are at the fore of all three of these battlefronts in the fight against COVID-19. Recently, therapeutic mAbs have become essential tools to defeat various diseases, including virus infections, based on their abilities to prevent disease progression immediately after administration and to speed up recovery, regardless of whether the patient has fully developed immunity [11].

SARS-CoV-2 is a single-stranded RNA virus belonging to the *betacoronavirus* genus. As with other viruses in this genus, several critical points in the life cycle of SARS-CoV-2 can potentially be targeted and blocked by mAbs, making mAbs promising prophylactic and therapeutic agents for COVID-19. The first critical point is when the virus S protein binds to a host cell receptor, such as ACE2 [12] or cluster of differentiation 147 (CD147) [13]. After the initial binding event, host proteases, such as furin, transmembrane serine protease 2 (TMPRSS2) and cathepsin L, cleave the head of S protein, transforming it into a spring-like structure; this action allows the viral membrane to fuse with the host membrane and enables direct cell surface entry or via endosome by endocytosis [14, 15]. Once the virus enters the host cell, its RNA is translated and the innate immune response is immediately induced via host expression of type I/III interferon, chemokines and cytokines, such as tumor necrosis factor (TNF), interleukin 1 beta (IL-1β), interleukin 6 (IL-6), and granulocyte-macrophage colony-stimulating factor (GM-CSF) [6, 16, 17]. Upon continued viral replication, the cytokine levels may keep rising, leading to severe tissue damage and cytokine release syndrome (CRS) in some patients [18]. Thus, therapeutic Abs that inhibit the biological activities of cytokines may alleviate the harmful effects of over-stimulated host immune response and serve as treatments for COVID-19 [19–23].

More than half of all people with SARS-CoV-2 infection have no symptoms; however, they may still be contagious in the asymptomatic state [24–26]. Four SARS-CoV-2 variants of concern that emerged in the United Kingdom (Alpha, B.1.1.7), South Africa (Beta, B.1.351), Brazil (Gamma, P.1) and India (Delta, B.1.617.2), have rapidly become dominant around the world and appear to display enhanced transmissibility and higher in-hospital mortality rates [27]. Moreover, B.1.1.529 was recently named Omicron and designated as a fifth variant of concern and by WHO after its emergence in South Africa [28]. Even more distressing, some other new SARS-CoV-2 variants that originally appeared in California (Epsilon, B.1.427 and B.1.429), Nigeria (Eta, B.1.525), New York (Iota, B.1.526), and India (Kappa, B.1.617.1 and Delta, B.1.617.2) are not only more transmissible but also exhibit reduced neutralization by convalescent and post-vaccination sera [29]. Thus, in addition to vaccines and therapeutic Abs, effective and rapid diagnostic tests for SARS-CoV-2 variants are necessary for timely medical and public health decisions, such as who should be placed in quarantine or hospitalized to reduce uncontrolled transmission. Molecular tests based on viral antigens can be used to identify individuals with acute phase SARS-CoV-2 infection, as well as control transmission when used in contact tracing, and allow for repeat testing in disease screening. Tests using Ab-antigen-formatted immunocomplexes are perhaps the most promising tools to accomplish this type of wide surveillance and control outbreaks of COVID-19. In this review, we summarize current knowledge about the use of neutralizing mAbs for prophylaxis, treatment and viral detection for COVID-19, especially focusing on those mAbs that are prime clinical candidates and have received emergency use authorization (EUA). We also describe how antibodies (Abs) can neutralize the virus in terms of S protein binding and structure. Finally, we propose strategies to combat the SARS-CoV-2 pandemic using therapeutic antibodies to overcome possible resistance of currently identified and potential mutants. The summarized information also provides insights into how therapeutic antibodies may be used against variants of SARS-CoV-2 in potential future pandemics.

## Therapeutic Abs

Currently, the global effects of COVID-19 continue to grow, and the disastrous pandemic requires fast development and implementation of countermeasures. To address these needs, researchers around the world are racing to develop therapies and vaccines. Among the technologies under intensive development, neutralizing mAbs are expected to be especially useful in prophylactic and therapeutic applications, based on the success of previously developed mAb drugs [30–33].

### Neutralizing Abs targeting spike (S) protein

**Fig. 1:**
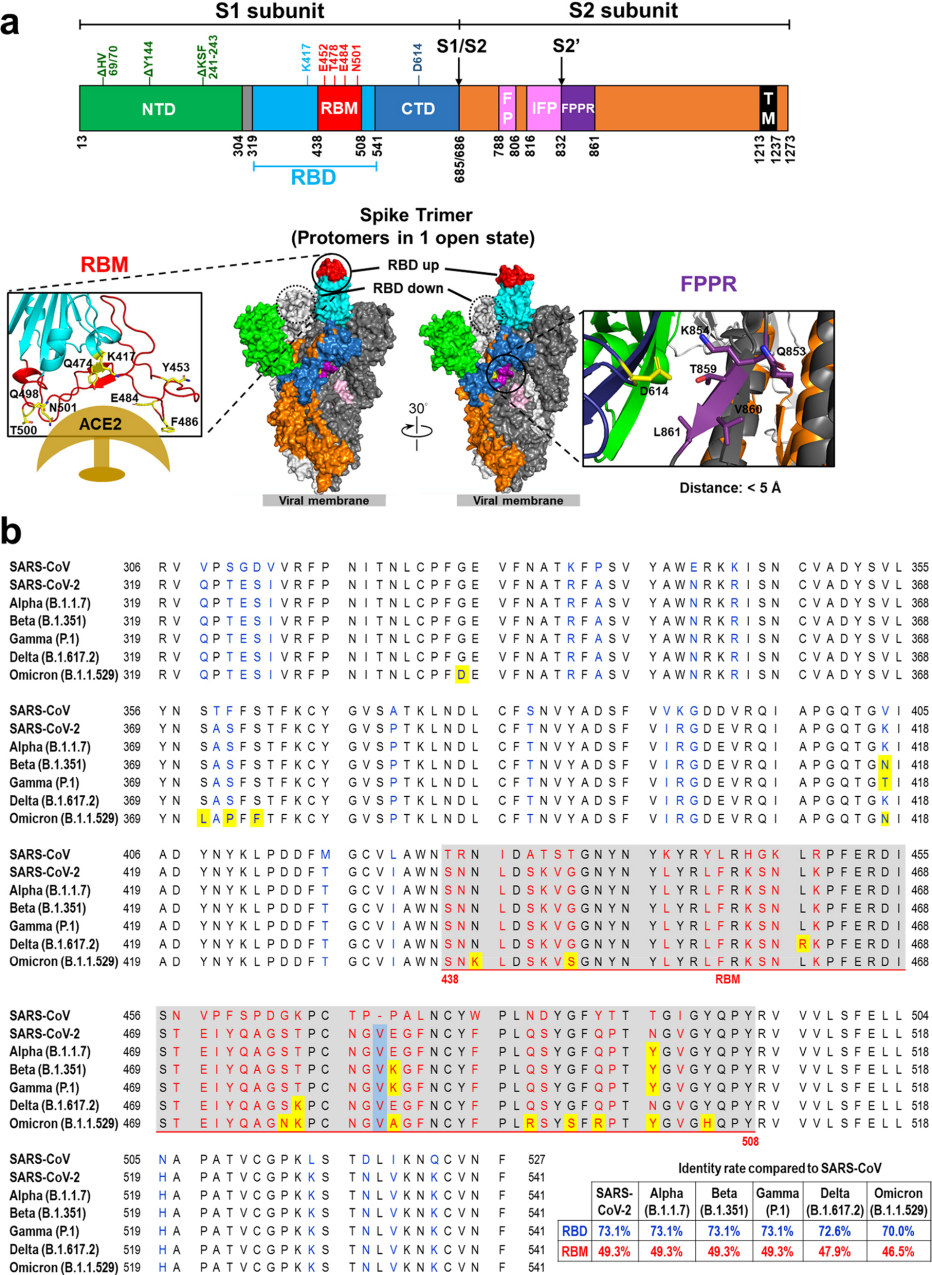
SARS-CoV-2 Spike protein. **a** Structure SARS-CoV-2 spike protein. Different domains of the SARS-CoV-2 spike protein: N-terminal domain (NTD), receptor-binding domain (RBD), receptor-binding motif (RBM), subdomain 1 and 2, protease cleavage sites (S1/S2/S2′), fusion peptide (FP), internal fusion peptide (IFP), fusion peptide proximal region (FPPR), and transmembrane region (TM). HV69/70, Y144, and KSF241-243 are frequently deleted residues in the NTD of SARS-CoV-2 variants of concern. K417, E452, E484, T478, N501 and D614 are the most frequently mutated residues in the RBD of SARS-CoV-2 variants of concern. Key residues of the receptor-binding motif in the S protein of SARS-CoV-2 that interact with ACE2 are shown (lower left). The SARS-CoV-2 S protein trimeric complex is shown in a “one-up” RBD conformation. The two RBD-down protomers are depicted in light and dark gray. The RBD-up protomer is colored according to its domains; RBM in red, non-RBM RBD in light blue, NTD in green, S2 in orange, FP and IFP in pink, and FPPR in purple. The dashed circle indicates the RBD site of an RBD-down conformation protomer. Inter-atomic contacts between aspartate 614 (yellow) in a reference S monomer (dark blue) and five residues (purple) in its adjacent S protein monomer chain (dark gray) within 5 Å. These five contacts might be destabilizing and create a hydrophilic-hydrophobic repulsion that is lost upon replacement of aspartate by glycine in the D614G mutation (lower right). **b** RBD sequences of SARS-CoV (GeneBank: AAP30030.1), SARS-CoV-2 (GeneBank: QVW76257.1), and SARS-CoV-2 variants of concern, including B.1.1.7 (Alpha), B.1.351 (Beta), P.1 (Gamma), B.1.617.2 (Delta), and B.1.1.529 (Omicron). The amino acids encoded by SARS-CoV-2 that are altered in comparison to SARS-CoV are colored blue (RBD) or red (RBM). The amino acid inserted in SARS-CoV-2 is denoted by a light blue background. The amino acids substituted in variants of concern are denoted by a yellow background. The residues 438–508 comprise the RBM of SARS-CoV-2 and are shown with grey background

The SARS-CoV-2 S protein is a trimeric complex that is cleaved into S1 and S2 subunits (Fig. 1a). S1 is responsible for receptor binding, while S2 is responsible for membrane fusion. On human cells, the S protein targets ACE2, a key regulator of the renin-angiotensin system, which acts as the cell entry receptor for the virus. S1 protein contains an N-terminal domain (NTD) and a receptor binding domain (RBD), which interacts with the peptidase domain of ACE2 through a receptor-binding motif (RBM). Although the role of the NTD is not entirely clear, it may be responsible for the recognition of specific sugar moieties upon initial attachment; such recognition could facilitate the transition of S protein from a prefusion state to a postfusion conformation. Abs binding to certain epitopes on the NTD have been shown to inhibit SARS-CoV-2 infection [34, 35]. Moreover, SARS-CoV-2 infectivity may also be enhanced by specific antibodies against the NTD, and infectivity-enhancing antibodies have been detected in severe COVID-19 patients [36]. Neutralization of S protein function has drawn considerable attention as a means to disrupt viral entry, making the S protein the most common target for new vaccines and drugs against SARS-CoV-2.

**Fig. 2:**
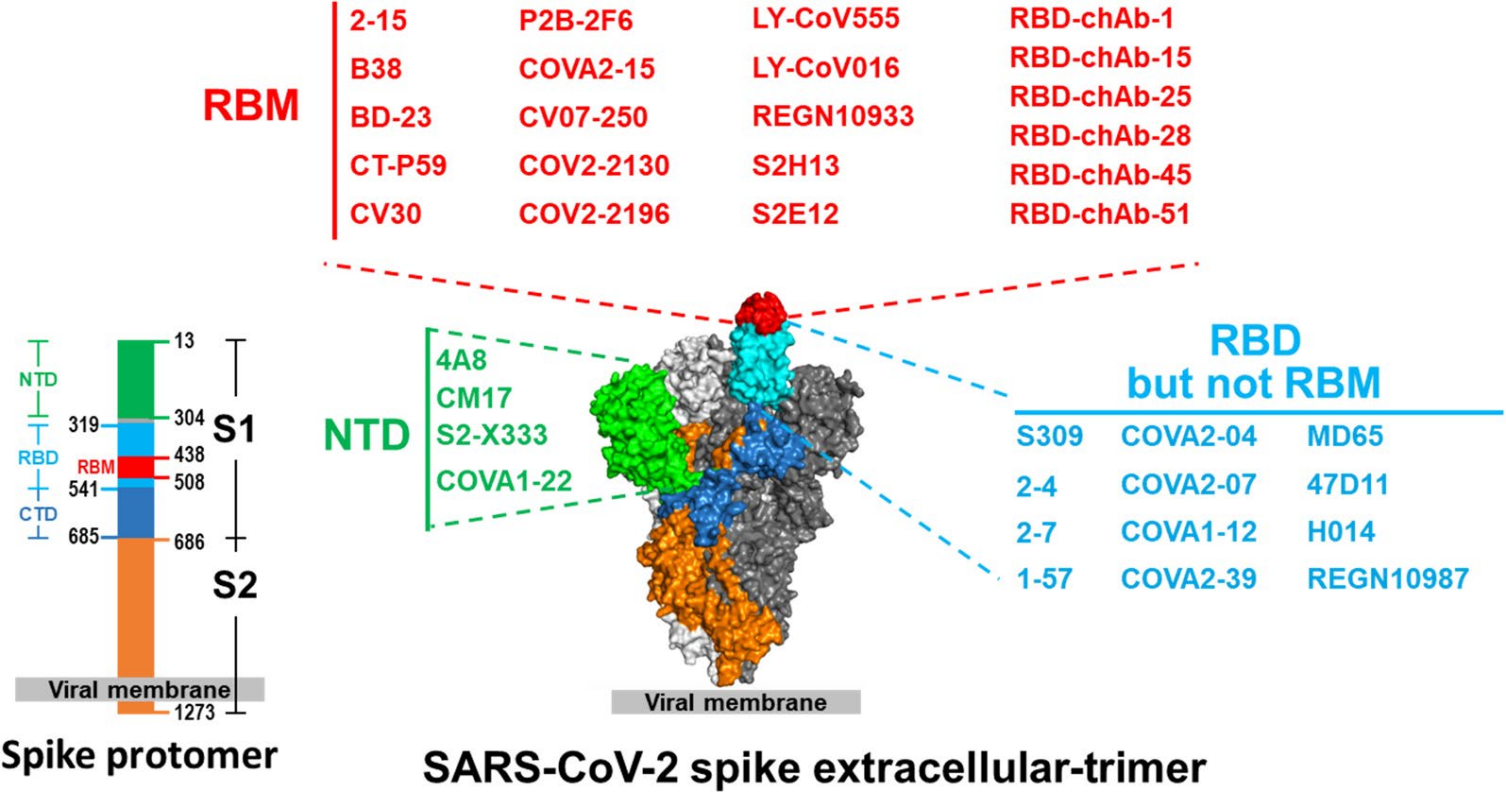
Epitopes of anti-spike and anti-RBD nAbs mapped to a surface model of SARS-CoV-2 spike trimer. The identified epitope regions are depicted as surface regions (PDB: 6VSB). Some of the shown anti-spike nAbs have known exact epitopes; for others the exact epitopes are unknown. Ab names are color-coded by the domains they recognized: N-terminal domain (NTD), light green; receptor binding motif (RBM), red; and receptor binding domain (RBD) but not RBM, cyan

An abundance of new SARS-CoV-2 S protein-specific mAbs have been reported by different researchers [32, 33, 37] and many bind to the RBD (Fig. 2, Table 1). The major strategy used for rapid isolation of high-efficacy nAbs is reverse transcriptase-polymerase chain reaction (RT-PCR) from single human B cells [38, 39]. In this approach, the SARS-CoV-2 S or RBD protein-specific memory B cells from convalescent or acute-phase COVID-19 patients are sorted by flow cytometry, and single-cell RT-PCR for immunoglobulin genes is performed. Alternatively, nAbs have also been generated using human Ab transgenic mice [40–43], phage display library screening [44–48], yeast surface display library screening [49] or hybridoma and Ab engineering technology [50].

**Table 1:** Summary of published SARS-CoV-2-neutralizing Abs until October, 2021

	Ab name*/epitope	Source	Method for structure	In vitro neutralization	In vivo experiment	References
1	B38/RBM	B cell, COVID-19 patient	X-ray crystallography	AV, CPE_50_ = 177.0 ng/ml	hACE2 mice, Treatment, 25 mg/kg, ↓3.3 log	[72]
2	47D11/RBD	Hybridoma mice, SARS-CoV	Cryo-EM	AV, PRNT_50_ = 570.0 ng/ml	Hamsters, Prophylactic, 3 mg/1 mL or 500 μL human convalescent plasma, TCID_50_ (Lung) ↓ 1–2 log	[40, 67, 246]
3	S309 (VIR-7831, Sotrovimab)/RBD	Human patient, SARS-CoV, and Single B cell	Cryo-EM	AV, FRNT_50_ = 79.0 ng/ml	Hamsters, Prophylactic, 5 mg/kg, viral load↓3 log	[96, 97]
4	311mab-31B5/RBD 311mab-32D4/RBD	B cell, COVID-19 patient	N.D	PSV, 311mab-31B5, IC_50_ = 33.8 ng/ml. 311mab-32D4, IC_50_ = 69.8 ng/ml	N.D	[100]
5	BD-23/RBM and N165 glycan of the neighboring “down” RBD	B cell, COVID-19 patient	Cryo-EM	AV, PRNT_50_ = 15.0 ng/ml	hACE2 mice, Prophylactic, 20 mg/kg, viral load, ↓ 7 log Treatment, 20 mg/kg, viral load, ↓ 4 log	[194]
6	2B04/RBD 1B07/RBD	Immunized Mouse single B cell	N.D	AV, 2B04, FRNT_50_ = 1.46 ng/ml 1B07, FRNT_50_ = 37.0 ng/ml	hACE2 mice, Prophylactic, 10 mg/kg, RNA 10^7^ → 10^6^	[247]
7	REGN10933(Casirivimab)/RBM REGN10987 (Imdevimab)/RBD	REGN10933/Humanized mice REGN10987/Patient single B cell	HDX-MS Cryo-EM	AV, REGN10933, PRNT_50_ = 5.6 ng/ml REGN10987, PRNT_50_ = 6.3 ng/ml	REGN10933 + REGN10987, Rhesus macaques, Prophylactic, 25 mg/kg, subgenomic RNA, ↓ 2 log Hamsters, Prophylactic, 50, 5, or 0.5 mg/kg, subgenomic RNA, ↓ 3, 2, 1 log Treatment, 50, 5, or 0.5 mg/kg, subgenomic RNA, ↓ 4, 4, 2 log	[41, 82]
8	4A8/NTD	B cell, COVID-19 patient	Cryo-EM	AV, virus RNA by qPCR IC_50_ = 390 ng/ml	N.D	[74]
9	COVA1-22/NTD COVA1-18/RBD COVA2-15/RBM	B cell, COVID-19 patient	Negative stain EM	PSV, COVA1-18, IC_50_ = 8.0 ng/ml COVA2-15, IC_50_ = 8.0 ng/ml AV, VeroE6 cells staining, COVA1-18, IC_50_ = 7.0 ng/ml COVA2-15, IC_50_ = 9.0 ng/ml	hACE2 mice, Prophylactic, 10 mg/kg, viral load, ↓ 4 log Treatment, 10 mg/kg, viral load, ↓ 4 log Hamsters, Treatment, 10 mg/kg, Viral titer, ↓ 3 log Cynomolgus macaques, Prophylactic, 10 mg/kg, absence of detectable sgRNA subgenomic RNA	[71, 248]
10	CV30/RBM	B cell, COVID-19 patient	N.D	PSV, IC_50_ = 30 ng/ml	N.D	[70, 249]
11	P2B-2F6/RBD	B cell, COVID-19 patient	X-ray crystallography	AV, P2B-2F6, PRNT_50_ = 50 ng/ml P2B-1F11, PRNT_50_ = 30 ng/ml	N.D	[250]
12	C121/RBD C135/RBD C144/RBD	B cell, COVID-19 patient	Negative stain EM, X-ray crystallography, and Cryo-EM	AV, VeroE6 cells infection (IFA) C121, IC_50_ = 1.64 ng/ml C135, IC_50_ = 2.98 ng/ml C144, IC_50_ = 2.55 ng/ml	N.D	[197, 251]
13	COV2-2130 (Cilgavimab)/RBM COV2-2196 (Tixagevimab)/RBM	B cell, COVID-19 patient	Negative stain EM	AV, FRNT COV2-2130, IC_50_ = 107 ng/ml COV2-2196, IC_50_ = 15 ng/ml PSV, COV2-2130, IC_50_ = 1.6 ng/ml COV2-2196, IC_50_ = 0.7 ng/ml	hACE2 mice, 10 mg/kg Prophylactic, lung plaque assay (PFU) COV2-2130, ↓ 3 log COV2-2196, ↓ 3 log COV2-2130 + COV2-2196, ↓ 3 log Rhesus macaques, 50 mg/kg Prophylactic, subgenomic viral RNA COV2-2196, ↓ 3 log BALB/c mice, 20 mg/kg Treatment, lung plaque assay (PFU) COV2-2130, ↓ 1 log COV2-2196, ↓ 4 log COV2-2130 + COV2-2196, ↓ 4 log	[100, 101]
14	IgG1 ab1/RBD	Fab, scFv, V_H_ phage display libraries	N.D	PSV, Luciferase reporter virus IC_50_ = 200 ng/ml	hACE2 mice, 3 mg/kg Prophylactic, lung plaque assay (PFU) 10^4.5^ → 10^1^	[39]
15	rRBD-15/RBD	Phage display	N.D	PSV, IC_50_ = 1830 ng/ml	N.D	[252]
16	HbnC3t1p1_C6/RBD	B cell, COVID-19 patient	N.D	AV, CPE, IC_100_ = 40 ng/ml	N.D	[253]
17	2–15/RBM 2–7/RBD	B cell, COVID-19 patient	Cryo-EM	AV, CPE 2–15, IC_50_ = 0.7 ng/ml 2–7, IC_50_ = 3.0 ng/ml PSV, CPE 2–15, IC_50_ = 5.0 ng/ml 2–7, IC_50_ = 10.0 ng/ml	Hamsters, 0.3 ~ 1.5 mg/kg 2–15, Prophylactic, RNA copy 10^6^ → 10^2^, ↓ 4 log	[66, 254]
18	S2H13/RBM	B cell, COVID-19 patient	Cryo-EM	PSV, IC_50_ = 500 ng/ml	N.D	[255]
19	S2M11/RBD S2E12/RBM	B cell, COVID-19 patient	Cryo-EM	PSV, S2M11, IC_50_ = 2.1 ng/ml S2E12, IC_50_ = 2.3 ng/ml AV, Focus-forming assay S2M11, IC_50_ = 1.2 ng/ml S2E12, IC_50_ = 4.2 ng/ml	Hamsters, 1 mg/kg, Prophylactic, S2M11, TCID_50_ 10^5^ → 10^1^, ↓ 4 log S2E12, TCID_50_ 10^5^ → 10^1^, ↓ 4 log S2M11 + S2E12, TCID_50_ 10^5^ → 10^1^, ↓ 4 log 0.5 mg/kg, Prophylactic, S2M11 + S2E12, TCID_50_ 10^5^ → 10^3^, ↓ 2 log	[256]
20	CV07-209/N.D. CV07-250/RBM	B cell, COVID-19 patient	X-ray crystallography	AV, CV07-209, PRNT_50_ = 3.1 ng/ml CV07-250, PRNT_50_ = 3.5 ng/ml	Hamsters, CV07-209, 18 mg/kg Prophylactic, ↓ 4 ~ 5 log, Treatment, ↓ 3 ~ 4 log	[257]
21	P008_056/NTD	B cell, COVID-19 patient	Cryo-EM and X-ray crystallography	AV, CPE_50_ = 30 ng/ml	N.D	[258]
22	58G6/RBM 13G9/RBM	B cell, COVID-19 patient	Cryo-EM	AV, 58G6, PRNT_50_ = 6.0 ng/ml 13G9, PRNT_50_ = 9.2 ng/ml PSV, 58G6, IC_50_ = 4.0 ng/ml 13G9, IC_50_ = 5.9 ng/ml	hACE2 mice, 10 mg/kg, Prophylactic, PRNT_50_ ↓ 3 log	[207]
23	S1D7/RBD S3D8/RBD	Immunised Mouse	N.D	AV, VeroE6 cells infection (IFA) S1D7, IC_50_ = 405 ng/ml S3D8, IC_50_ = 139 ng/ml S1D7 + S3D8, IC = 200 ng/ml	N.D	[259]
24	Wang-C387/RBD Wang-C437/RBD	B cell, COVID-19 patient	N.D	AV, VeroE6 cells infection (IFA) Wang-C387, IC_50_ = 8.4 ng/ml Wang-C437, IC_50_ = 2.0 ng/ml PSV, Wang-C387, IC_50_ = 10.6 ng/ml Wang-C437, IC_50_ = 4.9 ng/ml	N.D	[260]
25	S2-X333/NTD	B cell, COVID-19 patient	Cryo-EM	AV, S2-X333, IC_50_ = 2.0 ng/ml	Hamsters, viral challenge Viral RNA copies/mg lung: 4 mg/kg, 10^6^ → 10^3^, ↓ 3 log TCID_50_/mg lung: 1 mg/kg, 10^4^ → 10^1^, ↓ 3 log 4 mg/kg, 10^4^ → 10^1^, ↓ 3 log	[34]
26	C601/RBD	B cell, COVID-19 patient	Cryo-EM	PSV, Luciferase assay IC_50_ = 2.0 ng/ml	N.D	[191]
27	LY-CoV555 (Bamlanivimab)/RBD	B cell, COVID-19 patient	Cryo-EM and X-ray crystallography	AV, PRNT_50_ = 20 ng/ml (WA isolate) PRNT_50_ = 49 ng/ml (Italy isolate) PSV, stably transfected ACE2 IC_50_ = 12 ng/ml	Rhesus macaques, 2.5 mg/kg Prophylactic, BAL viral replication (Day3): ↓ > 1 log RNA copies/ml BAL viral replication (Day6): ↓ > 2 log RNA copies/ml Lung viral replication (Day6): ↓ > 3 log RNA copies/ml	[89]
28	XG003/RBD	B cell, COVID-19 patient	N.D	AV, XG005, IC_50_ = ~ 100 ng/ml XG014, IC_50_ = 5.1 ng/ml PSV, Luciferase assay XG005, IC_50_ = 6.1 ng/ml XG014, IC_50_ = 14.4 ng/ml	N.D	[261]
29	CM17/NTD	B cell, COVID-19 patient	Cryo-EM	AV, IC_50_ = 30 ng/ml	MA10 mice, virus titer (PFU), 10^5^ to 10^3^, ↓ 2 log (MA10 mice: BALB/c mouse model, a pathogenic mouse ACE2-adapted SARS-CoV-2 variant)	[262]
30	ABP18/RBD	Phage Display (Ab, human, non-immune)	N.D	PSV, Luciferase assay IC_50_ = 60 ng/ml	N.D	[263]
31	ION-360/RBD	B cell, COVID-19 patient	X-ray crystallography	PSV, Luciferase assay IC_50_ = 25.5 ng/ml	N.D	[264]
32	STE90-C11/RBD	Phage Display Library (Antibody, human, immune—CoV2)	X-ray crystallography	AV, PRNT_50_ = 84 ng/ml	N.D	[48]
33	FC05/NTD	Phage Display Library (Antibody, human, immune—CoV2)	Cryo-EM	N.D	N.D	[265]
34	P17/RBD	Phage Display (Ab, human, non-immune)	Cryo-EM	PSV, IC_50_ = 24.8 ng/ml, AV, PRNT_50_ = 29.2 ng/ml	hACE mice, 20 mg/kg Prophylactic + Treatment, ↓ 1.93 log RNA copies/g, > 2 log PFU/ml (lung) Treatment, ↓ 1 log RNA copies/g, > 2 log PFU/ml (lung)	[266]
35	HB27/RBD	Humanized from Immunised Mouse	Cryo-EM	PSV, IC_50_ = 6 ng/ml AV, PRNT_50_ = 33 ng/ml	hACE mice, 20 mg/kg Prophylactic, Day3 (lung): ↓ 5 log RNA copies/g, > 3 log PFU/ml Day5 (lung): ↓ 3 log RNA copies/g, > 1 log PFU/ml Therapeutic treatment: Day3 (lung): ↓ 4 log RNA copies/g, > 3 log PFU/ml Day5 (lung): ↓ 3 log RNA copies/g, > 1 log PFU/ml	[267]
36	6D3/S1-S2 cleavage Site	Mouse Hybridoma	X-ray crystallography	N.D	N.D	[268]
37	P4A1/RBD	B cell, COVID-19 patient	X-ray crystallography	PSV, IC_50_ = 975 ng/ml	Cynomolgus monkeys, 10 mg/kg, Day7 (lung): ↓ 3–4 log viral load (copies/g)	[43]
38	P5A-1B9/RBM	B cell, COVID-19 patient	Cryo-EM	AV, IC_50_ = 16.5 ng/ml PSV, IC_50_ = 12.0 ng/ml	N.D	[269]
39	TAU-1109/RBD	B cell, COVID-19 patient	N.D	PSV, pseudo-typed GFP SARS-CoV-2 IC_50_ = 45 ng/ml	N.D	[270]
40	58G6/RBD	B cell, COVID-19 patient	N.D	AV, RT-qPCR IC_50_ = 9.98 ng/ml	N.D	[271]
41	H014/RBD	Immunized Humanized (hACE2) Mouse	Cryo-EM	AV, PRNT_50_ = 5725.5 ng/ml	hACE2 mice, 50 mg/kg, Viral load, Treatment ↓ 1 log, Prophylactic + therapeutic treatment ↓2 log	[46]
42	BD-368–2/RBM	B cell, COVID-19 patient	Cryo-EM	AV, IC_50_ = 15 ng/ml: PSV, IC_50_ = 1.2 ng/ml	hACE2 mice, 20 mg/kg, Prophylactic, Viral load ↓6 log Treatment, Viral load ↓3 log	[194, 272]
43	CnC2t1p1_B4/RBD	B cell, COVID-19 patient	N.D	AV, IC_100_ = ~ 10,000 ng/ml	N.D	[253]
44	413–2/non-RBD	B cell, COVID-19 patient	N.D	AV, IC_50_ = ~ 7500 ng/ml PSV, IC_50_ = 8198 ng/ml	N.D	[273]
45	EY6A/RBD	B cell, COVID-19 patient	X-ray crystallography	AV, PRNT_50_ ~ 10,800 ng/ml	N.D	[60]
46	Fab1-20/RBD	B cell, COVID-19 patient	N.D	PSV, IC_50_ = 8 ng/ml	N.D	[66]
47	MD65/RBD	Phage Display Library (Antibody, human, immune—CoV2)	N.D	AV, PRNT_50_ = 220 ng/ml	N.D	[73]
48	CC12.1/RBD	B cell, COVID-19 patient	X-ray crystallography	PSV, HeLa-ACE2, IC_50_ = 46 ng/ml VER0-6, IC_50_ = 120 ng/ml	Hamsters, Prophylactic, 16.5 ~ 4.2 mg/kg Viral RNA, ↓2.5 log	[39, 274]
49	CA521/RBD	Transgenic Mouse	Cryo-EM	AV, PRNT_50_ = 0.73 ng/ml PSV, IC_50_ = 0.1 ng/ml	C57BL/6 mice, 20 mg/kg, Prophylactic, Viral RNA ↓2–4 log	[275]
50	BG10-19/RBD	B cell, COVID-19 patient	Cryo-EM and X-ray crystallography	PSV, D614G, IC_50_ = 2.0 ng/ml B.1.1.7, IC_50_ = 1.0 ng/ml B.1.351, IC_50_ = 4.0 ng/ml	N.D	[276]
51	COV2-2531/S2	B cell, COVID-19 patient	Negative stain EM	PSV, IC_50_ = 1.6 ng/ml	hACE2 mice, 10 mg/kg, Viral RNA ↓2 log	[207]
52	RBD-chAb-15/RBM RBD-chAb-25/RBM RBD-chAb-45/RBM	Hybridoma screening and humanized	Cryo-EM	AV, WT, RBD-chAb-15, PRNT_50_ = 30.3 ng/ml RBD-chAb-25, PRNT_50_ = 15.8 ng/ml RBD-chAb-45, PRNT_50_ = 9.9 ng/ml B.1.617.2, RBD-chAb-15, PRNT_50_ = 37.8 ng/ml RBD-chAb-45, PRNT_50_ = 18.0 ng/ml RBD-chAb-15 + 45, PRNT_50_ = 37.5 ng/ml PSV, WT, RBD-chAb-15, IC_50_ = 52.3 ng/ml RBD-chAb-25, IC_50_ = 25.44 ng/ml RBD-chAb-45, IC_50_ = 2.3 ng/ml B.1.617.2, RBD-chAb-15, PRNT_50_ = 103.6 ng/ml RBD-chAb-45, PRNT_50_ = 15.5 ng/ml RBD-chAb-15 + 45, PRNT_50_ = 25.7 ng/ml	Hamsters, Prophylactic**,** WT, 3 mg/kg RBD-chAb-15: TCID_50_ ↓ 1 log RBD-chAb-45: TCID_50_ ↓ 3.5 log RBD-chAb-15 + 45: TCID_50_ ↓ 4 log WT, 4.5 mg/kg RBD-chAb25: TCID_50_ ↓ 2 log RBD-chAb45: TCID_50_ ↓ 2 log RBD-chAb25 + 45: TCID_50_ ↓ 4 log AAV-hACE2 mice, Treatment, WT, 3 mg/kg RBD-chAb25 + 45: TCID_50_ ↓ 1.5 log Hamsters, Treatment, WT, 3 mg/kg RBD-chAb-15 + 45: TCID_50_ ↓ 4 log RBD-chAb25 + 45: TCID_50_ ↓ 4 log B.1.617.2, 6 mg/kg RBD-chAb-45: TCID_50_ ↓ 3 log RBD-chAb-15 + 45: TCID_50_ ↓ 3.5 log	[50, 75, 76]
53	CT-P59	B cell, COVID-19 patient	X-ray crystallography	AV, PRNT_50_ = 8.4 ng/ml	Ferrets, Treatment, 30 mg/kg TCID_50_ (Lung) ↓ 1 log Hamsters, Treatment, 30 mg/kg TCID_50_ (Lung) ↓ 7 log Rhesus monkeys, Treatment, 45 mg/kg TCID_50_ (Lung) unchanged	[47]
54	LY-CoV016 (Etesevimab, CB6 JS016,)/RBM	B cell, COVID-19 patient	X-ray crystallography	AV, CPE_50_ = 36 ng/ml PSV, CPE_50_ = 23 ng/ml	Rhesus monkeys, Prophylactic**,** 50 mg/kg Day3 (lung): ↓ 4 log RNA Rhesus monkeys, Treatment, 50 mg/kg Day3 (lung): ↓ 2 log RNA	[51]
55	2C08/RBD	B-cell; SARS-CoV-2 Vaccinee	Cryo-EM	AV, FRNT_50_ = 5 ng/ml	Hamsters, 2 mg/animal Prophylactic, viral RNA ↓ 3–4 log	[277]
56	S2X259/RBD	B cell, COVID-19 patient	Cryo-EM	AV, S2X259, PRNT_50_ = 144.2 ng/ml PSV, IC_50_ = 212.3 ng/ml	Hamsters, B.1.351 viral challenge TCID_50_/mg lung: 1 mg/kg, 10^4^ → 10^3^, ↓ 1 log 4 mg/kg, 10^4^ → 10^1^, ↓ 3 log 1 + 1 mg/kg with S309, 10^4^ → 10^1^, ↓ 3 log	[61]
57	A23-58–1/RBD	B cell, COVID-19 patient	Cryo-EM and Negative stain EM	AV, CPE, USA-WA1, IC_50_ = 2.0 ng/ml PSV, Luciferase assay D614G, IC_50_ = 1.8 ng/ml B.1.1.7, IC_50_ < 0.6 ng/ml B.1.351, IC_50_ = 1.6 ng/ml	N.D	[278]
58	COV107-23/RBD	B-cells; SARS-CoV-2 Human patient	X-ray crystallography	N.D	N.D	[279]
59	910–30/RBD	B-cells; SARS-CoV-2 Human patient	Cryo-EM	AV, CPE, IC_50_ = 183 ng/ml PSV, Luciferase assay IC_50_ = 66 ng/ml	N.D	[280]
60	DH1043/RBD DH1052/NTD	B-cells; SARS-CoV-2 Human patient	Cryo-EM and Negative stain EM	PSV, Luciferase assay DH1043, IC_50_ = 34 ng/ml DH1050, IC_50_ > 100,000 ng/ml	BALB/c mice, 30 mg/kg, Prophylactic, DH1052, viral RNA, ↓ 1 log Macaque, Prophylactic, 10 mg/kg lung subgenomic RNA DH1043, ↓ 5 log DH1052 ↓ < 1 log	[281]
61	C1A-B3/RBD	B-cells; SARS-CoV-2 Human patient	X-ray crystallography	AV, PRNT_50_ = 62 ng/ml PSV, Lentivirus pseudotype D614G, IC_50_ = 81 ng/ml	N.D	[282]
62	S2H97/RBD	B-cells; SARS-CoV-2 Human patient	X-ray crystallography and Cryo-EM	AV, PRNT_50_ = 794 g/ml PSV, PRNT_50_ = 338 ng/ml	Hamsters, 25 mg/animal Prophylactic, viral RNA ↓ 4 log	[62]
63	47D1/RBD	B-cells; SARS-CoV-2 Human patient	X-ray crystallography	AV, PRNT_50_ = 12.7 ng/ml PSV, Luciferase assay IC_50_ = 6.0 ng/ml	Hamsters, Prophylactic, 100, 25, or 6.25 mg/kg, lung viral RNA ↓ 1 log 1.6, or 0.4 mg/kg, lung viral RNA without difference	[283]
64	S2P6/S2	B-cells; SARS-CoV-2 Human patient	X-ray crystallography and Cryo-EM	PSV, Luciferase assay D614G, IC_50_ ~ 10,000 ng/ml P.1, IC_50_ ~ 10,000 ng/ml B.1.1.7, IC_50_ ~ 100,000 ng/ml B.1.351, IC_50_ ~ 100,000 ng/ml B.1.617.1, IC_50_ ~ 20,000 ng/ml	Hamsters, Prophylactic, Prototypic SARS-CoV-2 20 mg/kg, TCID_50_ (Lung) ↓ 2 log 2 mg/kg, TCID_50_ (Lung) < 1 log B.1.351 SARS-CoV-2 20 mg/kg, TCID_50_ (Lung) ↓ 1.5 log	[284]
65	P5A-3C8/RBD	B-cells; SARS-CoV-2 Human patient	X-ray crystallography	AV, FRNT_50_ = 11.2 ng/ml PSV, Luciferase assay IC_50_ = 20.6 ng/ml	Hamsters, Prophylactic, 5 mg/kg, lung viral RNA ↓ 1 log	[285]
66	5A6/RBD	Phage Display (Ab, human, non-immune)	Cryo-EM	AV, CPE, IC_50_ = 140.7 ng/ml PSV, Luciferase assay IC_50_ = 75.5 ng/ml	N.D	[286]
67	BLN12/NTD	Phage Display (Ab, human, immune [SARS-CoV-2])	N.D	AV, PRNT_50_ = 8.0 ng/ml	hACE2 mice, Prophylactic 5 mg/kg, 100% protection of death 0.5 mg/kg, 80% protection of death	[287]
68	N12-11/NTD	B-cells; SARS-CoV-2 Human patient	Cryo-EM	PSV, Luciferase assay IC_50_ ~ 490 ng/ml	N.D	[288]
69	2B11/RBD	Phage Display (Ab, human, immune [SARS-CoV-2])	X-ray crystallography	AV, PRNT_50_ = 1.0 ng/ml PSV, Luciferase assay IC_50_ = 6.0 ng/ml B.1.1.7, IC_50_ = 12.2 ng/ml B.1.351, IC_50_ = 5091 ng/ml P.1, IC_50_ = 2527 ng/ml	hACE2 mice, 25 or 75 mg/kg, Prophylactic, lung viral RNA ↓ 2 log Treatment, lung viral RNA ↓ 1 log	[289]
70	mAb40/RBD	B-cells; SARS-CoV-2 Human patient	N.D	AV, B.1.167.2, FRNT_50_ = 29 ng/ml PSV, Luciferase assay B.1.167.1, IC_50_ = 24 ng/ml B.1.167.2, IC_50_ = 24 ng/m B.1.1.519, IC_50_ = 17 ng/ml l B.1.429, IC_50_ = 11 ng/ml	N.D	[216]
71	C549/RBD	B-cells; SARS-CoV-2 Human patient	N.D	PSV, Luciferase assay WT, IC_50_ = 10.95 ng/ml Q493R, IC_50_ = 2.35 ng/m E484G, IC_50_ = 2.29 ng/ml R346S, IC_50_ = 8.33 ng/ml	N.D	[220]
72	SARS2-38/RBD	Immunised Mouse	Cryo-EM	AV, FRNT_50_ = 5.0 ng/ml PSV, FRNT_50_ = 6.0 ng/ml	hACE2 mice, 5 mg/kg Ab, Treatment, viral RNA, ↓ 3 log Hamsters, 10 mg/kg, Treatment, viral RNA, ↓ 2 log	[290]
73	54,042–4/RBD	B-cells; SARS-CoV-2 Human patient	Cryo-EM	PSV, Real-time cell analysis assay IC_50_ = 9.0 ng/ml AV, ELISA B.1.1.7, IC_50_ = 5.5 ng/ml B.1.351, IC_50_ = 9.7 ng/ml B.1.617.2, IC_50_ = 1.5 ng/ml P.1, IC_50_ = 3.7 ng/ml	N.D	[291]
74	MA1/RBD	Immunised Mouse	Cryo-EM	AV, Luciferase assay IC_50_ ~ 10 ng/ml PSV, Luciferase assay IC_50_ ~ 10 ng/ml	N.D	[292]
75	C12A2/RBD C12C9/NTD	B-cells; SARS-CoV-2 Human patient	Cryo-EM	AV, CPE USA-WA1 C12A2, IC_50_ = 2 ng/ml C12C9, IC_50_ = 43 ng/ml B.1.1.7, C12A2, IC_50_ = 8 ng/ml C12C9, IC_50_ = 6 ng/ml B.1.351, C12A2, IC_50_ > 50 ng/ml C12C9, IC_50_ > 500 ng/ml	N.D	[293]
76	TRES6/RBD	Transgenic Mouse	N.D	AV, CoV-2-ER1 (D614G) TRES6, IC_50_ = 102 ng/ml TRES6hu, IC_50_ = 33 ng/ml	hACE2 mice, viral challenge 5.25 mg/kg Ab, Log_10_ viral load (RNA copies) reduction: 4 days post, lung 30x, BAL 40x 10 days post, lung 100x, BAL 400x Prevented body weight loss, Reduced clinical symptoms	[294]
77	C1027/RBD	B-cells; SARS-CoV-2 Human patient	N.D	PSV, after 12 month WT, IC_50_ = 20.8 ng/ml K417N, IC_50_ = 4.1 ng/m E484K, IC_50_ = 3.4 ng/ml N501Y, IC_50_ = 16.8 ng/ml AV, after 12 month WA1/2020, IC_50_ = 9.35 ng/ml B.1.351, IC_50_ = 6.08 ng/ml	N.D	[295]
78	NT-193/RBD	Immunised mouse (TC-mAb)	X-ray crystallography	PSV, WT IgG1, IC_50_ = ~ 5.0 ng/ml IgG3, IC_50_ = ~ 1.0 ng/ml AV, WT, IgG1, TCID_50_ = ~ 600 ng/ml IgG3, TCID_50_ = ~ 600 ng/ml D614G, IgG1, TCID_50_ = ~ 250 ng/ml IgG3, TCID_50_ = ~ 150 ng/ml	Hamsters, IgG3, Viral RNA copies/mg lung: Prophylactic, 1.25 mg/kg, 10^6^ → 10^5^, ↓ 1 log 5 mg/kg, TCID_50_ 10^6^ → 10^5^, ↓ 1 log Treatment, 1.25 mg/kg, 10^6^ → 10^5^, ↓ 1 log 5 mg/kg, TCID_50_ 10^6^ → 10^4^, ↓ 2 log	[296]
79	7B8/RBD	Immunised mouse (RenMab)	Cryo-EM	**PSV,** D614G, IC_50_ = ~ 100 ng/ml B.1.1.7, IC_50_ = ~ 100 ng/ml N501Y, IC_50_ = ~ 100 ng/ml	N.D	[297]
80	CC40.1/RBD	B-cells; SARS-CoV-2 Human patient	X-ray crystallography	PSV, IC_50_ < 100 ng/ml	N.D	[298]
81	STE73-2E9/RBD	Phage Display Library (Antibody, human, immune-CoV2)	N.D	AV, TCID_50_ = 61.5 ng/ml	N.D	[48]
82	Fab-324/RBD	Phage Display Library (Antibody, human, non-immune)	Cryo-EM	PSV, Multabody, IC_50_ = 24 ng/ml IgG, IC_50_ = 21,000 ng/ml	N.D	[299]
83	P5C3/RBD	B-cells; SARS-CoV-2 Human patient	Cryo-EM	PSV, WT, IC_50_ = 4.0 ng/ml D614G, IC_50_ = 14.0 ng/ml E484K/N501Y, IC_50_ = 4.0 ng/ml K417N/E484K/N501Y, IC_50_ = 13.0 ng/ml AV, CPE WT, IC_50_ = 5.0 ng/ml D614G, IC_50_ = 11.0 ng/ml B.1.1.7, IC_50_ = 8.0 ng/ml B.1.351, IC_50_ = 3.0 ng/ml	Hamsters, Prophylactic, 5.0, 1.0, or 0.5 mg/kg Lung viral RNA, all 10^5^ → 10^2^, ↓ 3 log	[300]
84	PDI-222/RBD, WCSL-119/RBD	PDI-222: B-cells; SARS-CoV-2 Human patient WCSL-119: Semi-synthetic Human Fab Library	Cryo-EM	AV, PDI-222, WT, PRNT_50_ = 5.0 ng/ml D614G, PRNT _50_ = 11.0 ng/ml WCSL-119, WT, PRNT_50_ = 22.0 ng/ml D614G, PRNT _50_ = 25.0 ng/ml	B57BL mice, SARS-CoV-2 (D614G N501Y) Prophylactic, 5, 1, or 0.2 mg/kg PDI-222, TCID_50_ all ↓ 2 log WCSL-119**,** 5 or 1 mg/kg, TCID_50_ ↓ 2 log 0.2 mg/kg, TCID_50_ no change Hamsters, PDI-222, Prophylactic 5 mg/kg, TCID_50_ ↓ 5 log 0.25 mg/kg, TCID_50_ ↓ < 1 log	[301]
85	C1207/RBD	B-cells (Human Naïve, mRNA vaccination)	N.D	PSV, after 5 months vaccination WT, IC_50_ = 17.8 ng/ml K417N, IC_50_ = 7.2 ng/m N501Y, IC_50_ = 10.0 ng/ml E484K/R683G, IC_50_ = 3.3 ng/ml L525R/E484K/R683G, IC_50_ = 2.4 ng/ml	N.D	[302]

Note 1: In vitro neutralization experiment refers to authentic (AV) or pseudotyped (PSV) SARS-CoV-2 neutralization assay as indicated

Note 2: In vivo experiment refers to the animal type, Ab injected amount, and observed prophylactic or treatment efficacy as indicated

↓, decrease after compared to the control group; ~ , roughly estimated; n-log, n × 10 times; *AV* authentic SARS-CoV-2 virus, *CPE* cytopathic effect, *IC*_*50*_ half-maximal inhibitory concentrations, *IC*_*100*_ 100% inhibitory concentration, *IFA* immunofluorescence assays, *N.D.* not determined, *NTD* N-terminal domain, *PFU* plaque-forming unit, *PSV* SARS-CoV-2 pseudovirus, *PRNT*_*50*_ 50% reduction of plaque neutralization test, *qPCR* real-quantitative polymerase change reaction, *RBD* receptor binding domain, *RBM* receptor binding motif, *scFv* single-chain fragment variable, *TCID*_*50*_ median tissue culture infectious dose; ^*^Only listed representative Abs in indicated published papers

In order to screen Ab candidates for neutralizing capability in vitro, most groups test Abs against authentic living SARS-CoV-2, while some use pseudovirus with reporter readouts. A few methods have been used to quantify inhibitory concentrations, such as plaque reduction neutralization test (PRNT), focus reduction neutralization assay (FRNT), cytopathic effect (CPE), luciferase luminescence quantification, immunofluorescence assay (IFA), and virus mRNA quantification by quantitative polymerase change reaction (qPCR). The use of such a wide variety of in vitro assay methods makes it difficult to directly compare Abs from different publications (Table 1). To bring nAbs one step closer to clinical trials, a handful of publications also include data from in vivo animal models, which demonstrate the efficacy of the Ab as a treatment or prophylactic agent. Mice are not affected by SARS-CoV-2, presumably due to differences in ACE2 amino acid sequence compared to humans. Hence, mouse models for testing SARS-CoV-2 neutralizing capability must be generated by introducing human ACE2 into the lung cells of mice, either by the use of transgenic methods or by infecting normal mice with adenovirus encoding the human ACE2 gene for transient expression. As an alternative to mice, Shi et al. performed animal experiments in a rhesus macaque model; in this model, nAbs administered in both protection and treatment contexts caused clear reductions in viral load and lung damage [51]. Moreover, hamsters develop severe and easily observed signs of illness after infection with SARS-CoV-2, including rapid weight loss, a very high viral load in the lungs, and severe lung pathology [52]. Therefore, hamsters have become a commonly used model to evaluate the prophylactic and therapeutic efficacy of Abs (Table 1).

Next, we introduce prominent nAbs that bind to the RBD or non-RBD sites on the S protein, focusing on Abs that have received EUA from the U.S. FDA [53–58].

#### Neutralizing Abs targeting the S protein RBD

The RBD of the S protein is a target of multiple nAbs that inhibit SARS-CoV-2 infection by disrupting the interaction between the RBD and ACE2. Notably, the RBD sequence of SARS-CoV-2 S protein shares 73% amino acid identity with that of SARS-CoV (Fig. 1b), and the two viruses both possess a conserved epitope in the RBD that allows for possible Ab cross-reactivity. However, most SARS-CoV-nAbs do not bind the SARS-CoV-2 RBD, nor do they neutralize SARS-CoV-2 [59]. Only a few Abs have been shown to bind both SARS-CoV and SARS-CoV-2 [40, 60–62]. Researchers have used cryogenic electron microscopy (cryo-EM) to reveal that the structure of the SARS-CoV-2 S protein is an asymmetric trimer, with two conformations for the RBD (“open” and “closed”) [63, 64]. This dynamic conformation of the RBD may be a key factor affecting the neutralizing potency of anti-RBD Abs.

##### H014

Abs against the SARS-CoV-2 RBD were identified by screening a phage-display single-chain fragment variable (scFv) library generated from spleen mRNA of mice immunized with recombinant SARS-CoV RBD [46]. Among the hits from this screen, a potent nAb, H014, was found to bind the RBDs of SARS-CoV-2 and SARS-CoV with extremely high affinities (sub-nM concentrations). Cryo-EM reconstruction showed that H014 recognizes a conformational epitope on one side of the open (up) RBD, distinct from the RBM, whereas the closed RBD is inaccessible to H014. The authors had previously established human ACE2 knock-in mice using CRISPR/Cas9 technology as a model for SARS-CoV-2 infection [65]. The hACE2-humanized mice were infected with 5 × 10^5^ PFU of SARS-CoV-2 intranasally and then treated by intraperitoneal injection of H014 at 50 mg/kg. In therapeutic and prophylactic plus therapeutic groups, H014 treatment reduced viral titers in the lungs at day 5 by approximately tenfold and 100-fold, respectively. These results indicated a potential therapeutic use for H014 in treating COVID-19.

##### 2-15

Dr. David D. Ho’s group reported a collection of 61 SARS-CoV-2-nAbs from five infected patients with high plasma virus-neutralizing titers [66]. Their strategy for isolating Abs included sorting of SARS-CoV-2 S-specific memory B cells by flow cytometry and single-cell sequencing. Nineteen of the reported Abs could neutralize SARS-CoV-2 in vitro, with nine exhibiting high potency. Epitope mapping showed that about half of the 19 Abs are directed against the RBD, while the other half target the NTD, the top region of S protein. The RBD-directed Abs were shown to neutralize authentic SARS-CoV-2 virus with IC_50_ values of 0.7 to 209 ng/ml; the most potent Abs were 2-15, 2-7, 1-57 and 1-20. The NTD-directed Abs showed similar neutralizing activities, with the most potent being 2-17, 5-24 and 4-8 Abs. Cryo-EM structures were determined for several of the mAbs in complex with the S trimer to clarify Ab epitopes. The 2-4 Ab targeted the RBD and lock it into a “down” conformation, also obstructing the interaction with ACE2. The 4-8 Ab recognized the tip of the NTD, and 2-43 Ab recognized the top of the RBD, bridging two separate RBDs. In a study to evaluate prophylaxis in SARS-CoV-2-infected hamster models, a dosage of 1.5 mg/kg 2-15 showed protective efficacy, as it could reduce virus titer by more than four orders of magnitude. Thus, a relatively modest dose of this Ab almost completely prevented infection of SARS-CoV-2 in vivo.

Unfortunately, SARS-CoV-2 variants B.1.1.7 (Alpha) and B.1.351 (Beta) are resistant to neutralization by most NTD-targeting Abs, including 2-17, 5-24, and 4-8 [53]. However, both 5-24, and 4-8 retain the ability to inhibit the P.1 (Gamma) variant from Brazil [54]. Anti-RBD Abs (i.e., 2-15, 1-20 and 2-43) have impaired function against B.1.1.7 (Alpha), and the neutralizing potency against B.1.351 (Beta) is fully lost. The activity of anti-RBD Ab 1-57 is diminished by 1.5-fold against B.1.1.7 (Alpha) and 5.2-fold against B.1.351 (Beta). Meanwhile, the activity of 2-7 is unaffected by the variations in B.1.1.7 (Alpha), B.1.351 (Beta) and P.1 (Gamma), but its IC_50_ is reduced 3.4-fold when used against the E484K-single mutation pseudovirus [53, 54].

##### 47D11

Wang et al. characterized a human mAb, 47D11, which is capable of neutralizing both SARS-CoV and SARS-CoV-2 in vitro [40]. This Ab was generated from H2L2 human Ab transgenic mice, which were immunized with the S ectodomain of HCoV-OC43, SARS-CoV, and MERS-CoV. Cryo-EM structures showed that 47D11 binds specifically to the closed conformation of the RBD, distal to the ACE2 binding site [67]. Interestingly, 47D11 preferentially recognizes the partially open conformation of the SARS-CoV-2 S protein, suggesting that it could be used effectively in combination with other Abs that target the exposed RBM. AbbVie has a license for this Ab from Harbour BioMed and completed a phase I clinical trial for the prevention and treatment of COVID-19 [68, 69].

##### CV30

Hurlburt et al. isolated a potent neutralizing mAb, CV30, from a patient infected with SARS-CoV-2 [70]. CV30 binds the RBD, neutralizes pseudovirus with an IC_50_ of 0.03 μg/ml, and competes for binding sites with ACE2. The X-ray crystal structure revealed that CV30 almost exclusively binds to the RBM in the RBD. Notably, CV30 has minimal somatic mutations compared to the germline sequence; it has only a two-residue change in heavy chain of variable domain and no change in the light chain of variable domain.

##### COVA2-15

Brouwer et al*.* isolated 19 nAbs from three convalescent COVID-19 patients using a stabilized prefusion SARS-CoV-2 S protein [71]. These Abs target a diverse range of epitopes on the S protein, and two showed picomolar neutralizing activities against authentic SARS-CoV-2 virus. EM was used to reveal the structures of six RBD antigen-binding fragments (Fabs). Four interacted with a stoichiometry of one Fab per trimer, with RBDs in the up state. COVA2-15 was able to bind RBD domains in both the up and down states.

##### B38 and H4

Wu et al. isolated four nAbs from a convalescent COVID-19 patient. Two of the Abs, B38 and H4, blocked RBD binding to ACE2 [72]. The *K*_*d*_ for B38 binding to the RBD was measured using surface plasmon resonance (SPR) at 70.1 nM, while that of H4 was 4.48 nM. The abilities of B38 and H4 Abs to protect against SARS-CoV-2 in vivo were also explored. hACE2 transgenic mice were treated with a single dose of 25 mg/kg B38 or H4 Abs 12 h after viral challenge. The RNA copies of virus in both the B38-treated and H4-treated groups were significantly reduced (by 3.3 and 2.7 orders of magnitude, respectively). A competition assay indicated the B38 and H4 epitopes on the RBD are different, and a cocktail of both Abs exhibited synergistic neutralizing ability in Vero-E6 cells. This pair of Abs could therefore potentially be used together to prevent immune escape in clinical applications.

##### MD65

Phage display is a powerful technique that enables rapid, efficient, and high-throughput selection of Abs (scFv or Fab) against antigens in vitro [48]. Several human Ab drugs derived from phage display libraries have been approved and are currently on the market. Noy-Porat et al. constructed a phage display scFv library using peripheral circulatory lymphocytes collected from patients in the acute phase of disease [73]. The phage scFv library complexity was 9.2 × 10^6^, and the library was used for affinity selection of Abs against RBD-human fragment crystallizable (Fc). Eight fully human, SARS-CoV-2-nAbs were isolated and characterized. These Abs target four distinct epitopes on the S protein RBD. Evaluation of the Ab affinities toward S1 by biolayer interferometry (BLI) revealed *K*_*d*_ values of these human Abs ranging from 0.4 to 5.8 nM. The neutralization potencies of the Abs were then evaluated by PRNT using VeroE6 cells infected with the SARS-CoV-2. MD65 displayed the highest neutralization potency with a PRNT_50_ concentration of 0.22 μg/ml.

##### 4A8

Chi et al. identified three neutralizing mAbs from 10 convalescent COVID-19 patients [74]. Among these mAbs, 4A8 exhibits high neutralization potency against authentic SARS-CoV-2. Interestingly, however, 4A8 does not bind the RBD. Cryo-EM was used to determine the structure of 4A8 in complex with the S protein, revealing that its epitope is located in the NTD of S protein, and that the Ab binds to S1 with *K*_*d*_ of 92.7 nM. 4A8 exhibits moderate neutralizing capacity, with an EC_50_ of 0.61 μg/ml, but it does not block the binding of S protein to the ACE2 receptor. Thus, 4A8 functions via a mechanism that is independent of receptor binding inhibition. According to the structure of the complex, the mechanisms of neutralization may involve restraining conformational changes in S protein.

##### RBD-chAb-1, 15, 25, 28, 45 and 51

In a recent study, a panel of Abs against the SARS-CoV-2 RBD were generated from mouse hybridoma Ab screening and were engineered into human immunoglobulin G (IgG)1 chimeric Abs [50]. Among these Abs, six potent nAbs, RBD-chAb-1, 15, 25, 28, 45, and 51, were found to bind the RBD of SARS-CoV-2 with high affinities (K_D_ values lower than 6.5 × 10^–9^ M) and high neutralizing activities (PRNT_50_ values lower than 10 ng/ml). Experiments using site-directed mutagenesis and competition-binding assays further indicated that these six chAbs bind to three distinct epitopes within the RBM. Cryo-EM reconstruction was then used to show that the epitopes of two highly potent Abs, RBD-chAb-25 and 45, are on one side of the open (up) RBD. This structural analysis suggested that RBD-chAb-25 and 45 can simultaneously bind to the same RBD, and the simultaneous binding was confirmed by size-exclusion chromatography. Importantly, the prophylactic effects of these Abs were demonstrated in an AAV-hACE2 mouse model and a hamster model, and the cocktail of RBD-chAb-25 and 45 showed highly promising therapeutic effects [50]. Notably, several antibody cocktails showed low IC_50_ values (3.35–27.06 ng/ml) against the SARS-CoV-2 variant pseudoviruses including Alpha, Beta, Gamma, Epsilon, Iota, Kappa and Delta variants [75]. Furthermore, the therapeutic treatment with an antibody cocktail of RBD-chAb-15 and 45 effectively protected hamsters from infection with the Delta SARS-CoV-2 variant [75].

Yang et al. further identified a unique salt bridge switch involving the B.1.1.7 (Alpha)-specific A570D mutation. The RBD-up state is stabilized by a double salt bridge involving A570D-K854 and D571-K964. Thus, introduction of the A570D mutation to S protein with D614G should lead to increased sensitivity of the virus to three RBD-up-specific Abs. Furthermore, the combined use of RBD-chAb-15 and 45, which simultaneously bind to distinct regions of the RBD, is also an attractive strategy for a prophylactic cocktail to prevent mutational viral escape [76].

#### EUA anti-SARS-CoV-2 therapeutic Abs

As of December 2021, the number of mAbs targeting S protein that were under evaluation in clinical trials was 25 (Table 2). At least 27 countries and 274 companies/institutions are developing Ab therapeutics [77], and these Abs have been comprehensively described in several review papers [33, 55, 56, 77–80]. Up to now, only seven Abs, including bamlanivimab, etesevimab, casirivimab, imdevimab, sotrovimab, cilgavimab and tixagevimab have been approved or received EUAs from the U.S. FDA (Table 2). In the following paragraphs, we introduce and update information regarding the development of these Ab treatments.

**Table 2:** Clinical studies evaluating anti-SARS-CoV-2 mAbs

No.	Name	Start date	Latest Status	Developer	Country	References
1	LY-CoV555 (Bamlanivimab)	5/28/2020	EUA (11/09/2020) EUA revoked (4/9/2021)	Eli Lilly/AbCellera	Canada/USA	NCT04411628, NCT04427501, NCT04497987, NCT04501978, NCT04518410 [89]
2	LY-CoV555 (Bamlanivimab) + LY-CoV016 (Etesevimab)	6/17/2020	EUA (2/09/2021)	Eli Lilly/AbCellera/Junshi	Canada/USA	NCT04427501, NCT04497987 [94]
3	REGN-COV2 (REGN10933/Casirivimab + REGN10987/Imdevimab)	6/10/2020	EUA (11/21/2020) Approved (8/10/2021)	Regeneron	USA	NCT04425629, NCT04426695, NCT04452318 [41, 55, 81, 82]
4	S309 (VIR-7831, Sotrovimab)	8/27/2020	EUA (5/26/2021)	Vir biotechnology/ GlaxoSmithKline	USA/UK	NCT04501978, NCT04545060 [96]
5	AZD7442 (COV2-2130/Cilgavimab + COV2-2196/Tixagevimab)	8/17/2020	EUA (12/08/2021)	AstraZeneca/Vanderbilt University Medical Center	UK/USA	NCT04501978, NCT04507256, NCT04625725, NCT04625972
6	TY027	6/09/2020	Phase III	Tychan Pte. LTD	Singapore	NCT04429529, NCT04649515 [303]
7	BRII-196 + BRII-198	7/12/2020	Phase III	Brii Bio/TSB Therapeutics/Tsinghua University	China/USA	NCT04518410, NCT04501978 [250]
8	CT-P59 (Regdanvimab)	7/18/2020	Phase II/III EUA (South Korea)	Celltrion	South Korea	NCT04525079, NCT04593641, NCT04602000 [47]
9	BI 767551 (DZIF-10c)	11/23/2020	Phase II/III	University of Cologne/he German Center for Infection Research/Boehringer Ingelheim	Germany	NCT04631705, NCT04631666, NCT04822701 [253]
10	SCTA01	7/24/2020	Phase II/III	Sinocelltech Ltd/Chinese Academy of Sciences	China	NCT04483375, NCT04644185 [46]
11	ADG20	4/26/2021	Phase II/III	Adagio Therapeutics	USA	NCT04805671, NCT04859517
12	MAD0004J08	3/1/2021	Phase II/III	Toscana Life Sciences Sviluppo s.r.l	Italia	NCT04932850, NCT04952805
13	MW33	8/7/2020	Phase II	Mabwell (Shanghai) Bioscience	China	NCT04533048
14	DXP593	8/31/2020	Phase II	Beigene	China	NCT04532294, NCT04551898 [194]
15	COVI-AMG (STI-2020)	2/2/2021	Phase II	Sorrento Therapeutics	USA	NCT04734860
16	LY-CoV1404 + LY-CoV555 (Bamlanivimab) + LY-CoV016 (Etesevimab)	11/18/2020	Phase II	Eli Lilly/AbCellera/ Junshi	USA	NCT04634409
17	XVR011	5/12/2021	Phase I/II	Exevir Bio BV	Belgium	NCT05017168
18	LY-CoV016 (JS016, Etesevimab)	6/5/2020	Phase I	Junshi Biosciences/ Chinese Academy of Sciences/Eli Lilly	China/USA	NCT04441918, NCT04441931, NCT04427501 [51]
19	47D11	11/25/2020	Phase I	Utrecht University/Abbvie/Erasmus MC/Harbor BioMed	Netherlands/China/USA	NCT0464412 [40]
20	ADM03820	12/4/2020	Phase II/III	Ology Bioservices	USA	NCT04592549, NCT05142527
21	DXP604	12/15/2020	Phase I	Beigene	China	NCT04669262
22	C144-LS and C-135-LS	1/11/2021	Phase II/III	Bristol-Myers Squibb, Rockefeller University	USA	NCT04700163, NCT04518410 [98]

##### REGN-COV2 (casirivimab and imdevimab)

REGN-COV2 is a cocktail of the human Abs, casirivimab and imdevimab (formerly known as REGN10933 and REGN10987, respectively), which both target the S protein RBD but were identified by different methods [41]. Casirivimab was identified from VelocImmune hAb transgenic mice immunized with a DNA plasmid encoding SARS-CoV-2 S protein, followed by a booster of injected recombinant S protein. Meanwhile, imdevimab was identified from isolated PBMCs of three human donors previously infected with SARS-CoV-2. In both cases, the murine or human single B cells bound to S protein were sorted by FACS. The *K*_*d*_ values of casirivimab and imdevimab for S protein are both about 0.04 nM by measurement with Biacore T200. The PRNT_50_ of casirivimab and imdevimab are 0.0374 and 0.0421 nM, respectively. Novel S gene mutants rapidly appeared when virus was passaged in the presence of individual Abs, resulting in loss of neutralization. However, treatment of casirivimab and imdevimab together can prevent the selection of escape mutants in vitro since they comprise a non-competing Ab cocktail [81]. In vivo efficacy of this Ab cocktail has been evaluated in both rhesus macaques (used to model mild disease) and golden hamsters (model for more severe disease) [82]. In the rhesus macaques, REGN-COV2 greatly reduced virus load in the lower and upper airways and decreased virus-induced pathological sequelae when administered prophylactically (50 mg/kg dosage) or therapeutically (25 mg/kg dosage). Administration in hamsters (5 mg/kg dosage) inhibited weight loss and reduced viral titers in the lung.

Four separate large clinical trials are ongoing for REGN-COV2. One of the trials is a phase I–III adaptive, randomized, placebo-controlled, double-blind trial (NCT04425629) on non-hospitalized patients with COVID-19, aiming to reduce the risk of treatment-resistant mutant virus emergence [55]. Seven hundred ninety-nine patients were randomly assigned (1:1:1) to receive placebo, 2.4 g of REGN-COV2, or 8.0 g of REGN-COV2. The interim analysis showed that REGN-COV2 can indeed reduce viral load in patients. Safety outcomes were similar in the combined REGN-COV2 dose groups and the placebo group. The above results supported the EUA designation for the casirivimab and imdevimab cocktail, which was granted by the U.S. FDA on November 20, 2020 for COVID-19 therapy. Under the EUA, the recommended dose is 1.2 g of casirivimab and 1.2 g of imdevimab (2.4 g total), administered as a single intravenous infusion. The phase III data showed that the combined casirivimab and imdevimab treatment could reduce the risk of COVID-19-related hospitalization and death by 70% COVID-19 in non-hospitalized patients, and the median time of symptom duration was reduced from 14 to 10 days.

In April 2021, new data from a phase III treatment trial in recently infected asymptomatic COVID-19 patients demonstrated that subcutaneous injection of a 1.2 g total dose of REGN-COV2 (1:1, casirivimab:imdevimab) reduced the risk of progression to symptomatic COVID-19 by 31%, and the risk was reduced by 76% after the third day. Furthermore, another positive result from a phase III COVID-19 prevention trial in uninfected household contacts of SARS-CoV-2 infected individuals showed that the 1.2 g total dose of REGN-COV2 reduced the risk of symptomatic infections by 81% [83]. REGN-COV2 was granted an EUA by the U.S. FDA in December 2020 and gained full approval from Japan’s Ministry of Health, Labour and Welfare in July 2021 for the treatment of patients with mild to moderate COVID-19 [84].

As casirivimab and imdevimab were designed against the SARS-CoV-2 strains that were being transmitted at the beginning of the pandemic in 2020 [41, 81], there is some question as to the protective and therapeutic ability against newly emerged variant strains; however, the treatment remains effective or at least does not cause concern when used against new variants. Most recently, it has been reported that B.1.1.7 (Alpha) is not refractory to the neutralizing activity of casirivimab and imdevimab [53]. Notably, the B.1.351 (Beta) and P.1 (Gamma) variants are fully resistant to casirivimab and slightly resistant to the neutralization by imdevimab [53, 54]. However, the combination of casirivimab and imdevimab show prophylactic and therapeutic efficacy against SARS-CoV-2 variants including viruses with B.1.1.7 (Alpha), B.1.351 (Beta), or P.1 (Gamma) in animals [85]. With regard to the newly emerged B.1.617.2 (Delta) variant, casirivimab also exhibits reduced neutralizing ability; however, imdevimab and the cocktail of casirivimab and imdevimab can still efficiently block virus S protein entry into the host cell [86]. Moreover, according to the REGN-COV2 fact sheet authorized by the U.S. FDA, pseudovirus assays showed that the neutralizing activity of REGN-COV2 was not changed with regard to currently circulating variants, including B.1.1.7 (Alpha), B.1.351 (Beta), P.1 (Gamma), B.1.429 (Epsilon), and B.1.526 (Iota). On August 10, 2021, the U.S. FDA authorized REGN-COV2 for both treatment and post-exposure prophylaxis (prevention) of COVID-19; the approved dosage is 600 mg of casirivimab and 600 mg of imdevimab administered together [87].

In January 2021, the US government signed a contract to purchase 1.25 million doses of REGN-COV2, and is expected to pay US$2.625 billion to Regeneron ($2,100/dose). The company anticipates being able to provide at least 1 million doses by June 30, 2021 if the EUA is updated to the lower 1,200 mg dose. The European Medicines Agency (EMA) also approved the use of REGN-COV2 and stated that clinical results show that the use of REGN-COV2 treatment can reduce the amount of virus in the nose and throat of patients, thereby reducing the number of patient visits to health care providers. In January 2021, the German government purchased 200,000 doses at a price of US$488 million ($2,440/dose). In February 2021, the French government announced that it had distributed thousands of doses of REGN-COV2 to various hospitals for clinical treatment of patients. In May 2021, the governments of Belgium and Switzerland approved clinical use of REGN-COV2. Also in May, Japan completed an agreement with Roche to purchase REGN-COV2. Total sales for the first half of 2021 consisted of $4.156 billion for REGN-COV2 [88]

##### Bamlanivimab (LY-CoV555)

Bamlanivimab is a human IgG1 targeting the RBD of S protein. It was discovered by Eli-Lilly and AbCellera via a high-throughput microfluidic screen of antigen-specific B cells from the first U.S. patient to recover from COVID-19 [89]. In a rhesus macaque challenge model, prophylactic doses as low as 2.5 mg/kg reduced viral replication in the upper and lower respiratory tract. On May 28, 2020, a clinical trial for bamlanivimab was initiated on hospitalized patients with COVID-19, and the Ab became the world’s first SARS-CoV-2-specific Ab to be used for COVID-19 therapy.

In the phase II trial of Blocking Viral Attachment and Cell Entry with SARS-CoV-2 Neutralizing Antibodies (BLAZE-1; NCT04427501), 452 patients with mild to moderate COVID-19 were randomly assigned to receive a single intravenous infusion of bamlanivimab at one of three doses (700 mg, 2800 mg, or 7000 mg) or placebo; patients were evaluated for quantitative virologic endpoints and clinical outcomes [57]. Those patients treated with bamlanivimab showed reduced viral load and lower rates of symptoms and hospitalization. Based on data from the BLAZE-1 study, the U.S. FDA granted an EUA for a single infusion of 700 mg bamlanivimab for the treatment of mild to moderate COVID-19 in adults and pediatric patients on November 9, 2020 [56]. Thus, bamlanivimab was the first SARS-CoV-2-nAb authorized for clinical use. Eli Lilly has an agreement with the U.S. government to supply 300,000 vials of 700 mg doses of bamlanivimab for US$375 million ($1250/dose) [90]. According to Eli Lilly, the company plans to donate COVID-19 therapies to Direct Relief for use in low- and lower-middle-income countries, which have been heavily impacted by the pandemic.

There is some concern that while bamlanivimab activity is unaffected against the B.1.1.7 (Alpha) variant strain, its protective efficacy is lost against the B.1.351 (Beta), P.1 (Gamma), and B.1.617.2 (Delta) variants, due to the E484 mutation [53, 54, 85, 86]. The use of a yeast display library to comprehensively map mutations in the RBD that allow SARS-CoV-2 to escape Ab binding [91] revealed that the L452R mutation in the B.1.429 (Epsilon) lineage allows escape from bamlanivimab [92]. Because emerging data shows that common SARS-CoV-2 viral variants are resistant to bamlanivimab alone, the U.S. FDA revoked the EUA that allowed for bamlanivimab to be used as a monotherapy of COVID-19 patients on April 9, 2021.

##### Combination of bamlanivimab with etesevimab

Etesevimab (CB6, JS016, LY-CoV016) was identified by screening single B cells from a convalescent patient [51]. X-ray crystallography revealed that its epitope on SARS-CoV-2 RBD largely overlaps with ACE2 binding residues. To reduce the potential risk of an Ab-dependent enhancement (ADE) [93] and effector functions, the Fc of etesevimab was modified by two leucine-to-alanine substitutions at residues 234 and 235 (known as the LALA mutation), which abolished its affinity for the Fcγ receptor. In rhesus monkey models, treatment with etesevimab inhibited viral titers and reduced lung damage under both prophylactic and therapeutic usages. Etesevimab has been evaluated in a completed phase I clinical trial (NCT04441931) and a phase II/III study in combination with bamlanivimab (NCT04427501).

On January 26, 2021, Eli Lilly announced that the combination of bamlanivimab (2.8 g) and etesevimab (2.8 g) significantly reduced hospitalizations and deaths in high-risk patients recently diagnosed with COVID-19, reaching the primary endpoint of the Phase III BLAZE-1 trial (NCT04427501). In the 1035 patients enrolled in this trail, the treatment reduced hospitalizations and death by 70%. There were 10 deaths in total, all of which occurred in patients taking placebo, and no deaths were recorded in patients taking bamlanivimab and etesevimab together. The Phase III BLAZE-1 trial showed additional results to demonstrate combination of bamlanivimab 700 mg and etesevimab 1400 mg reduced the risk of COVID-19 related hospitalizations and deaths by 87% in high-risk patients aged 12 and older and recently diagnosed with the virus. The data were from 769 high-risk patients with mild to moderate COVID-19. Of those patients, 511 were randomly assigned to treatment with Ab cocktail, and the other 258 were assigned to placebo. The primary endpoint was percentage of participants who experience COVID-related hospitalizations or death from any cause by day 29.

Based on the BLAZE-1 trial, the U.S. FDA issued an EUA for combined bamlanivimab (700 mg) and etesevimab (1400 mg) for the treatment of mild to moderate COVID-19 in patients of at least 12 years old who weigh at least 40 kg and are at high risk of progressing to severe disease and/or hospitalization. This combination therapy is expected to reduce the risk of selecting for resistant viruses when compared to bamlanivimab administered alone [94]. While the combination of bamlanivimab and etesevimab can neutralize B.1.1.7 (Alpha), it is not protective against B.1.351 (Beta) and P.1 (Gamma) variants because of the K417N/T mutation [53, 54]. Regarding the newly emerged B.1.617.2 (Delta) variant, bamlanivimab loses neutralizing ability due to the E484Q mutation, whereas etesevimab is not influenced by this mutation and still retains neutralizing ability. Therefore, the cocktail of bamlanivimab and etesevimab has partially reduced ability to inhibit B.1.617.2 (Delta) variant [86].

The U.S. government agreed to purchase up to 1.2 million doses of bamlanivimab and etesevimab together by November 2021. One hundred thousand doses have been ordered for shipment by March 31 at a value of US$210 million ($2,100 USD/dose). According to Eli Lilly's financial report for the first quarter of 2021, bamlanivimab and etesevimab had global sales of US$810 million, ranking first among all product lines. In May 2021, Eli Lilly plans to provide bamlanivimab and etesevimab to low- and middle-income countries free of charge. Bamlanivimab and etesevimab has begun to be used in India, and the first Indian patient treated with this Ab cocktail was discharged from the hospital in Haryana on May 26, 2021. The Medanta hospital in India reported that the cocktail is also effective against B.1.617.2 (Delta) variant and that the price of each dose is US$815. Lilly reported that total sales for the first half of 2021 consisted of $959.1 million for bamlanivimab and etesevimab administered together [88]. However, results from in vitro assays show that bamlanivimab and etesevimab administered together are not active against either the P.1 (Gamma) or B.1.351 (Beta) variants. Therefore, the U.S. Department of Health and Human Services paused all distribution of etesevimab alone, and bamlanivimab and etesevimab together on June 25, 2021 [95].

##### Sotrovimab (VIR-7831, S309)

Sotrovimab is a derivative of the S309 mAb, which was engineered with an extended half-life and potentially improved biodistribution in the lungs by the introduction of a LS mutation in the Fc [96]. S309 was originally identified from memory B cells of an individual with SARS-CoV infection in 2003; this Ab was found to potently cross-neutralize authentic SARS-CoV-2 [97]. Cryo-EM analysis revealed that S309 can bind to the “up” and “down” states of the RBD in a single S trimer. However, the Fab engages an epitope distinct from the RBM and does not compete with ACE2 upon binding to S glycoprotein. It was proposed that the mechanism of S309-mediated neutralization may be the induction of S trimer cross-linking, steric hindrance, or aggregation of virions. S309 also showed strong Ab-dependent cell cytotoxicity and Ab-dependent cellular phagocytosis effector functions. The Fc-effector function was demonstrated to contribute to the neutralization of SARS-CoV-2 in mouse models [98].

A phase III COVID-19 mAb Efficacy Trial (COMET-ICE) evaluated sotrovimab (0.5 g, intravenous injection) as a monotherapy for the early treatment of COVID-19 in adults at high risk of hospitalization. The study was stopped early in March 2021 due to clear evidence of clinical efficacy. Interim study results demonstrated an 85% reduction in the primary endpoint of hospitalizations (more than 24 h) or death for those receiving sotrovimab (n = 291) compared to placebo (n = 292). On May 26, 2021, the U.S. FDA issued an EUA for the 0.5 g single-dose of sotrovimab for the treatment of mild-to-moderate COVID-19 in pediatric patients (12 years of age and older) who are at high risk for progression to severe COVID-19. In vitro testing showed that sotrovimab retains activity against currently circulating variants, including P.1 (Gamma), B.1.429 (Epsilon), B.1.526 (Iota) and B.1.617.2 (Delta) [53, 54, 99].

##### AZD7442 (tixagevimab and cilgavimab)

AZD7442 is the combination of two human mAbs initially isolated from convalescent patients after SARS-CoV-2 infection and later engineered to be long-acting IgG molecules. The mAbs, COV2-2130 (AZD1061/cilgavimab) and COV2-2196 (AZD8895/tixagevimab), recognize and simultaneously bind to two distinct non-overlapping epitopes on the virus RBD in the “up” configuration [100, 101]. COV2-2130 and COV2-2196 both have neutralizing abilities, with IC_50_ values of 1.6 ng/mL and 0.7 ng/mL in pseudovirus assays, and IC_50_ values of 107 ng/mL and 15 ng/mL in FRNT, respectively (Table 1). Furthermore, a dose of 50 mg/kg showed a major protective effect in Rhesus macaques, with no subgenomic viral RNA detected in the treated group. By contrast, the isotype control mAb group had high levels of subgenomic viral RNA after exposure to SARS-CoV-2. In a mouse experiment to evaluate the therapeutic effects of the combination, 80% of treated mice had undetectable levels of infectious virus in lung after receiving the most effective dose of approximately 20 mg/kg [100]. AstraZeneca licensed the combination in June 2020, and the mAbs were then further optimized by modifying amino acid residues in the Fc region [102]. First, L234F/L235E/P331S substitutions in the Fc region mitigate the potential risk of FcγR and complement binding [103]. Second, M252Y/S254T/T256E substitutions were made to increase the affinity for human FcRn at low endosomal pH, extending the half-lives of the mAbs [104]. After optimization, a single dose of AZD7442 was shown to provide protection against COVID-19 for 6 to 12 months [102].

On 15 June 2021, AstraZeneca announced results from a phase III trial (STORM CHASER) assessing the safety and efficacy of AZD7442 for the prevention of symptomatic COVID-19 in participants recently exposed to the SARS-CoV-2. AZD7442 reduced the risk of developing symptomatic COVID-19 by 33% compared to placebo, which did not meet the primary endpoint. However, other phase III trials PROVENT and TACKLE are still ongoing and will evaluate the respective efficacies of AZD7442 for pre-exposure prevention and preventing severe disease. Most recently, it has been reported that the combination of COV2-2130 and COV2-2196 can neutralize SARS-CoV-2 variants, including B.1.1.7 (Alpha), B.1.351 (Beta), P.1 (Gamma), B.1429 (Epsilon), B.1617.1, or B.1526 (Iota), in vitro. From the analysis of prophylactic and therapeutic efficacies against B.1.1.7 (Alpha), B.1.351 (Beta), or P.1 (Gamma) in animals, AZD7442 showed promising results [105]. In November 2021, new data from two phase III trials testing AZD7442 for prophylaxis and post-exposure prophylaxis were released (Table 2). The 6-month follow-up of the prevention trial showed that one 300 mg IM (intramuscular injection) dose of AZD7442 reduced risk of symptomatic COVID-19 by 83%, with no severe disease or deaths observed. The separate treatment trial showed 88% reduced risk of severe COVID-19 or death when treatments were given within three days of symptom onset [106]. Based on this progress, AstraZeneca has already signed an agreement with the U.S. government to supply up to 500,000 doses of AZD7442 for US$205 million ($410/dose), contingent on AZD7442 receiving EUA in post-exposure prophylaxis [107].

### Antibodies to control the cytokine storm syndrome (CSS)

Cytokine storm syndrome (CSS) or CRS is an uncontrolled systemic inflammatory response associated with highly increased levels of inflammatory cytokines responding to different triggers, including therapies, pathogens or autoimmune disease. Critical COVID-19 patients often exhibit CSS-like syndromes, such as high fever, severe pneumonia leading to ARDS, multiple organ failure, or even death. Therefore, it is reasonable to suspect that the direct effects of CSS, triggered by exaggerated levels of inflammatory cytokines, are at least partially responsible for severe COVID-19 syndrome [108]. Although the role of these inflammatory factors in treatment of COVID-19 remains unclear, effectively neutralizing the overproduced inflammatory factors in CSS is essential to reduce mortality in patients with COVID-19 [109–111]. Here, we summarize the current clinical-stage therapeutic mAbs that can target cytokines to relieve CSS in COVID-19 patients (Table 3).

**Table 3:** Clinical trials of therapeutic antibodies for COVID-19

Target & mAb drug	ClinicalTrials.gov identification	Type	Phase
***Anti-IL-6***			
Clazakizumab	NCT04348500, 6 trials	Humanized rabbit IgG1 mAb	II
Siltuximab	NCT04329650, 3 trials	Chimeric IgGκ mAb	II/III
Olokizumab	NCT04452474, 2 trials	Humanized IgG4 mAb	II/III
***Anti-IL-6R***			
Levilimab	NCT04397562	Human mAb	III
Sarilumab	NCT04661527, 9 trials	Human IgG1 mAb	I/II/III
Sirukumab	NCT04380961	Human IgG1κ mAb	II
Tocilizumab	NCT04372186, 56 trials	Humanized mouse IgG1 mAb	EUA
***Anti-IL-1β***			
Canakinumab	NCT04362813, 5 trials	Human IgG1κ mAb	III
***Anti-TNF***			
Infliximab	NCT04425538, 4 trials	Chimeric IgG1 mAb	II
Adalimumab	NCT04705844	Human mAb	III
***Anti-GM-CSF***			
Lenzilumab	NCT04351152	Human IgG1 mAb	III
Otilimab	NCT04376684	Human IgG1 mAb	II
TJ003234	NCT04341116	Human IgG1 mAb	II/III
***Anti-GM-CSFR***			
Gimsilumab	NCT04351243	Human IgG1 mAb	II
***Anti-GM-CSFR-α***			
Mavrilimumab	NCT04447469, 5 trials	Human IgG4 mAb	II/III
***Anti-C5***			
Eculizumab	NCT04346797, 4 trials	Humanized mouse IgG2/4κ mAb	II
***Anti-C5a***			
Vilobelimab	NCT04333420	Chimeric IgG4 mAb	II/III
***Anti-C5aR***			
Avdoralimab	NCT04371367, 2 trials	Human IgG1 mAb	II
***Anti-PD-1***			
Nivolumab	NCT04356508, 3 trials	Human IgG4 mAb	II

#### Abs targeting interleukin-6 (IL-6)

The consistent observation of high IL-6 levels in CSS patients suggests that this cytokine is a key mediator of CSS, although the mechanisms of such action have not yet been fully elucidated [112]. IL-6 is known to be essential for the adaptive immune response in which T cells and B cells are recruited to the infected site. There are two main pathways of IL-6 signaling transduction, referred to as classic cis or trans signaling. In classic cis signaling, IL-6 and gp130 form a complex with membrane-bound IL-6 receptor (mIL-6R), while in the trans pathway, they bind to the soluble form of IL-6 receptor (sIL-6R). In either case, the IL-6 receptor (IL-6R) signaling complex activates intercellular signaling involved in a wide range of biological functions, such as immune regulation through downstream JAK-STAT3 signaling [113]. Importantly, elevated IL-6 level has been tightly associated with ARDS and high mortality of COVID-19 patients; therefore, IL-6 is thought to be a promising therapeutic target to reduce hyper inflammation and prevent the high mortalities of COVID-19 [112, 114–116]. According to the key role of IL-6 in CSS, several mAb drugs have been considered for the treatment of severe COVID-19, including sarilumab (Kevzara), tocilizumab (Actemra) and levilimab, which target IL-6R, as well as clazakizumab, siltuximab and olokizumab, which target IL-6 [19, 20, 117, 118].

These Abs specifically bind to both mIL-6R and sIL-6R and inhibit both cis and trans signal transduction. Several reports suggested that critically ill patients with COVID-19 who received tocilizumab or sarilumab had improved outcomes and lower rates of mortality [119, 120]. However, other studies on the efficacy of tocilizumab or sarilumab have shown conflicting results, as the drugs failed to reduce the risk of intubation or death in patients with COVID-19 in several clinical trials [118, 121–124]. Despite these inconclusive results, the U.S. FDA granted authorization for the emergency use of tocilizumab to treat patients hospitalized with COVID19 on June 24, 2021; the decision was based on the findings from a large clinical trial on tocilizumab [125, 126]. The EUA is specifically for treating certain hospitalized patients who are already receiving corticosteroids and need breathing support, but the drug is not approved as a general treatment for COVID-19. In the clinical trials on critically ill patients with COVID-19 in the intensive care unit, both tocilizumab and sarilumab improved survival [119, 127]. Furthermore, in clinical trials on hospitalized patients, tocilizumab used for the treatment of COVID-19 reduced the risk of death within 28 days by an absolute difference of 4% compared with usual care; this result was from patients with COVID-19 who required oxygen and had evidence of inflammation. Tocilizumab also reduced the time that patients remained in the hospital, and the probability of patient discharge within 28 days was raised from 50 to 57% (*p* < 0.0001) [125]. This trial provided the most definitive evidence that treatment with tocilizumab benefits hospitalized COVID-19 patients [120]. In addition, the WHO has recommended the use of tocilizumab and sarilumab plus corticosteroids to treat severe COVID-19 [127].

#### Targeting TNF

TNF is an important cytokine in many inflammatory diseases, and it is known to regulate IL-6 expression. In contrast to anti-IL-6 therapy, anti-TNF therapy has been shown to downregulate several inflammatory cytokines including IL-1, IL-6, and GM-CSF [128, 129]. Moreover, elevated levels of TNF in the blood and tissues of patients with COVID-19 have been indicated in previous reports [130]. Since blocking IL-6 met with limited success in COVID-19 patients, anti-TNF therapy has been recently considered as a means of reducing inflammation in COVID-19 [21, 131]. Early observations from clinical data support the idea that anti-TNF Abs, such as infliximab or adalimumab may reduce the mortality rate in patients with COVID-19 [132, 133]. Up to now, there have been four clinical trials on infliximab (NCT04344249, NCT04425538, NCT04593940, NCT04734678) and one on adalimumab (NCT04705844), all of which seek to evaluate their therapeutic potential in COVID-19.

#### Targeting IL-1β

There are three important cytokines in the IL-1 family that are especially relevant to cytokine storms: IL-1β, IL-18, and IL-33; among these cytokines, blocking IL-1β has great potential to counteract cytokine storms [22]. The IL-1 family members play different pro-inflammatory roles in patients with COVID-19, and these individual cytokines may be important mediators of many CSS symptoms, including fever, edema, and finally, organ dysfunction or death. Thus, blocking their function may possibly reverse the cytokine storm. Though the exact roles of IL-1 cytokines in the pathogenesis of CSS are unclear, it seems that IL-1 receptor blockade may help to maintain better control of inflammatory processes. Canakinumab is a human mAb that neutralizes IL-1β bioactivity by competing for IL-1RI binding; it is approved for the treatment of cryopyrin-associated periodic syndromes and several serious auto-inflammatory diseases [134, 135]. Clinical studies have been performed to examine the efficacy and safety of canakinumab in patients with COVID-19 [136, 137].

#### Others

Besides IL-1β, IL-6, and TNF, several cytokine storm-related factors are potential therapeutic targets for the treatment of severe COVID-19 patients. For example, GM-CSF is often found at a high level in COVID-19 patients. GM-CSF binding to GM-CSF receptor-α (GM-CSFR-α) stimulates IL-1, IL-6, and TNF production, promoting downstream Janus kinase 2 (JAK2) signal transduction [138]. Mavrilimumab is a human mAb targeted to GM‐CSFR-α that has been used as an investigational drug for the treatment of rheumatoid arthritis [139]. Recently, clinical data suggest that the condition of COVID-19 patients with pneumonia and systemic hyper inflammation can be improved by treatment of mavrilimumab and lenzilumab [23, 140, 141]. These results showed that therapeutic antibodies against GM-CSF can improve the clinical outcomes for COVID-19 patients with CSS.

In addition to GM-CSF, the complement system may be a valuable target for COVID-19 therapy, as it is an integral component of the innate immune response to virus infection. Complement signaling comprises three known axes, including the classical complement, alternative complement, and lectin pathways. All three pathways converge on the main component C3 of the complement pathway and result in the production of proinflammatory anaphylatoxins, C3a and C5a, and the formation of the terminal membrane attack complex (MAC) [142]. Patients with severe COVID-19 showed complement activation and high concentrations of C5a and MAC, suggesting that dysregulation of the complement pathway may participate in CSS and severe COVID-19 complications [143–147]. Notably, mechanistic studies showed the S or nucleocapsid protein of SARS-CoV-2 can activate the complement pathway [148, 149]. Based on the apparent involvement of complement in COVID-19, clinical studies have been initiated for several Abs, including avdoralimab, eculizumab, and vilobelimab (Table 3). Eculizumab is a humanized mAb with a high affinity to C5 that inhibits the generation of C5a and C5b proteins and prevents the formation of the inflammatory anaphylatoxin and the MAC [150]. In addition, avdoralimab and vilobelimab are mAbs targeting C5aR or C5a that prevent binding of C5a to C5aR and block the formation of the inflammatory anaphylatoxin associated with pulmonary pathology of ARDS in COVID-19 [145, 151]. Conceivably, these therapeutic antibodies could be effective treatments for severe COVID-19 with CSS.

## Antibody-based SARS-CoV-2 detection

As the number of patients with COVID-19 continues to grow around the world, a major issue is monitoring and evaluating patients with diagnostic tests that can distinguish SARS-CoV-2 from other viruses causing common cold symptoms [152]. Tests for viral nucleic acids and antigens can specifically indicate the presence of the virus in patients during the acute phase of virus infection [153]. Moreover, the diagnostic sensitivity of each test varies depending on the duration of disease, viral load, and quality of specimen collection, in addition to the collection site [154]. Because SARS-CoV-2 mainly replicates in the respiratory tract, the U.S. Centers for Disease Control and Prevention (CDC) recommends collecting and analyzing patient specimens from the upper and lower respiratory tract [155]. The nucleic acid detection assays show better sensitivity when used on specimens from the lower respiratory tract, including bronchoalveolar lavage fluid and sputum, than for specimens from the upper respiratory tract [156]. Among upper respiratory specimens, swabs collected from the nasal cavity yield a higher detection rate than oropharyngeal swabs [157].

In one of the main nucleic acid amplification tests, RT-PCR is used to amplify a unique viral genome sequence with specific primers; this assay offers a high accuracy and was the first method developed for SARS-CoV-2 detection, making it the gold standard [158]. The CDCs from several different countries have provided RT-PCR protocols using oligonucleotide primers and probes that are complementary to several regions of the SARS-CoV-2 genome, including N, E, replicase ORF1a and ORF1b [159]. However, RT-PCR is time-consuming and requires trained personnel with specialized equipment in the laboratory. Therefore, rapid and sensitive point-of-care testing (POCT) assays have been developed, including many based on lateral flow immunoassay (LFIA).

In contrast to detection of the virus, COVID-19 serology tests detect Abs that are produced as part of the human immune response to antigen from the pathogen. Seroconversion for immunoglobulin M (IgM) and IgG may occur simultaneously or sequentially [160]. For COVID-19, seroconversion of IgM and IgG are observed an average of 13 days after onset of symptoms [160]. Serology tests using whole blood, plasma, or serum-containing abundant immunoglobulins can reveal a patient’s medical history after infection, which is useful for demonstrating Ab kinetics or assessing vaccine effectiveness [161]. One study analyzed millions of individuals diagnosed with COVID-19, showing that people aged 65 or older had higher rates of reinfection with SARS-CoV-2 [162]. Especially with regard to such vulnerable populations, serology tests can be applied to identify pre-asymptomatic individuals with SARS-CoV-2 reinfection, control transmission when used in contact tracing, and allow for repeat testing in disease screening.

Immunoassays based on antigen-Ab interactions include enzyme-linked immunosorbent assay (ELISA), chemiluminescence immunoassay (CLIA), and LFIA; these assays are widely applied for detection of specific antigens or Abs related to infectious agents [163]. The major antigens used for serology tests are purified N and S proteins, which can be applied alone or in combination to generate immunoassays that broadly detect different isotypes of Ab (Table 4). The nucleoprotein (NP) binds and packs the viral RNA genome into a helical nucleocapsid for viral replication [164]. Meanwhile, the S protein plays a significant role in viral fusion and entry into host cells and is composed of S1 RBD at N-terminus and S2 subunits at C-terminus [165]. Previous reports indicated that both NP and S protein are immunogenic, as Abs against NP and the RBD of S protein as well as their B cell epitopes were readily detected upon early seroconversion in COVID-19 patients [166–169]. ELISAs using NP and RBD of S protein show high specificity and no cross-reactivity with non-CoV, HCoV, MERS-CoV, or SARS-CoV [170, 171]. Furthermore, a meta-analysis identified 38 studies that showed the use of RBD as an antigen provides higher sensitivity than NP [172]. In addition, the presence of RBD-specific Abs is also highly associated with COVID-19 nAb response [173–176].

**Table 4:** Serology tests with EUA from the U.S. FDA

Name	Company	Source	Target	Accuracy	Method
Alinity i SARS-CoV-2 IgG	Abbott	IgG	NP	IgG Sens 0–7 days: 49.3% 8–13 days: 80.4% ≥ 14 days: 98.1%, IgG Spec: 99.6%	CLIA
Architect SARS-CoV-2 IgG	Abbott	IgG	NP	IgG Sens 0–7 days: 49.3% 8–14 days: 82.6% ≥ 15 days: 98.1%, IgG Spec: 99.6%	CLIA
AdviseDx SARS-CoV-2 IgM (Architect)	Abbott	IgM	S	IgM Sens 0–7 days: 42.6% 8–14 days: 79% ≥ 15 days: 95%, IgM Spec: 99.6%	CLIA
Babson Diagnostics aC19G1	Babson Diagnostics, Inc	IgG	S	IgG Sens 8–14 days: 66.7% ≥ 15 days: 100% IgG Spec: 100%	CLIA
Access SARS-CoV-2 IgG	Beckman Coulter, Inc	IgG	S	IgG Sens 0–7 days: 75.8% 8–14 days: 95.3% ≥ 15 days: 96.8%, IgG Spec: 99.6%	CLIA
Access SARS-CoV-2 IgM	Beckman Coulter, Inc	IgM	S	IgM Sens 0–7 days: 54.4% 8–14 days: 91.7% ≥ 15 days: 98.3%, IgM Spec: 99.9%	CLIA
SARS-CoV-2 IgG and IgM Combo Test	BioCheck, Inc	IgM, IgG	S	IgM Sens 0–7 days: 100% 8–14 days: 93.8% ≥ 15 days: 88.9%, IgM Spec: 97.2% IgG Sens 0–7 days: 100% 8–14 days: 100% ≥ 15 days: 100%, IgG Spec: 100%	CLIA
SARS-CoV-2 IgG Antibody Test Kit	BioCheck, Inc	IgG	S	IgG Sens 0–7 days: 100% 8–14 days: 100% ≥ 15 days: 100%, IgG Spec: 100%	CLIA
SARS-CoV-2 IgM Antibody Test Kit	BioCheck, Inc	IgM	S	IgM Sens 0–7 days: 100% 8–14 days: 93.8% ≥ 15 days: 88.9%, IgM Spec: 97.2%	CLIA
LIAISON SARS-CoV-2 IgM Assay	DiaSorin, Inc	IgM	S	IgM Sens 0–7 days: 64.4% 8–14 days: 90.2% ≥ 15 days: 92.6%, IgM Spec: 99.3%	CLIA
LIAISON SARS-CoV-2 S1/S2 IgG	DiaSorin, Inc	IgG	S	IgG Sens 0–5 days: 25% 6–14 days: 89.8% ≥ 15 days: 97.55%, IgG Spec: 99.3%	CLIA
LIAISON SARS-CoV-2 TrimericS IgG	DiaSorin, Inc	IgG	S	IgG Sens 0–7 days: 21.4% 8–14 days: 70.8% ≥ 15 days: 96.9%, IgG Spec: 99.5%	CLIA
DZ-Lite SARS-CoV-2 IgG CLIA Kit	Diazyme Laboratories, Inc	IgG	S, NP	IgG Sens 0–7 days: 43.5% 8–14 days: 91.7% ≥ 15 days: 100%, IgG Spec: 97.4%	CLIA
DZ-Lite SARS-CoV-2 IgM CLIA Kit	Diazyme Laboratories, Inc	IgM	S, NP	IgG Sens 0–7 days: 26.1% 8–14 days: 83.8% ≥ 15 days: 94.4%, IgG Spec: 98.3%	CLIA
QUANTA Flash SARS-CoV-2 IgG	Inova Diagnostics, Inc	IgG	S, NP	IgG Sens 0–7 days: 66.7% 8–14 days: 61.5% ≥ 15 days: 100%, IgG Spec: 99.9%	CLIA
VITROS Anti-SARS-CoV-2 IgG test	Ortho-Clinical Diagnostics, Inc	IgG	S	IgG Sens 12–15 days: 83.3% ≥ 16 days: 90%, IgG Spec: 100%	CLIA
VITROS Immunodiagnostic Products Anti-SARS-CoV-2 Total Reagent	Ortho-Clinical Diagnostics, Inc	Pan-Ig	S	Pan-Ig Sens 0–7 days: 80% ≥ 8 days: 100%, Pan-Ig Spec: 100%	CLIA
Q-Plex SARS-CoV-2 Human IgG (4 Plex)	Quansys Biosciences, Inc	IgG	S	IgG Sens 0–7 days: 100% 8–14 days: 100% ≥ 15 days: 95.2%, IgG Spec: 99.7%	CLIA
MAGLUMI 2019-nCoV IgM/IgG	Shenzhen New Industries Biomedical Engineering Co., Ltd	IgM, IgG	S, NP	IgM Sens 0–7 days: 43.8% 8–14 days: 78.3% ≥ 15 days: 77.5%, IgM Spec: 99.6% IgG Sens 0–7 days: 31.3% 8–14 days: 90.6% ≥ 15 days: 100%, IgG Spec: 99.1%	CLIA
ADVIA Centaur SARS-CoV-2 IgG (COV2G)	Siemens Healthcare Diagnostics	IgG	S	IgG Sens 0–6 days: 53.5% 7–13 days: 93.4% ≥ 14 days: 100%, IgG Spec: 99.9%	CLIA
ADVIA Centaur SARS-CoV-2 Total (COV2T)	Siemens Healthcare Diagnostics	Pan-Ig	S	IgG Sens 0–6 days: 61.1% 7–13 days: 97.5% ≥ 14 days: 100%, IgG Spec: 99.8%	CLIA
Atellica IM SARS-CoV-2 IgG (COV2G)	Siemens Healthcare Diagnostics	IgG	S	IgG Sens 0–6 days: 56% 7–13 days: 92.2% ≥ 14 days: 100%, IgG Spec: 99.9%	CLIA
Atellica IM SARS-CoV-2 Total (COV2T)	Siemens Healthcare Diagnostics	Pan-Ig	S	Pan-Ig Sens 0–6 days: 60.7% 7–13 days: 97.5% ≥ 14 days: 100%, Pan-Ig Spec: 99.8%	CLIA
Vibrant COVID-19 Ab Assay	Vibrant America Clinical Labs	Pan-Ig	S, NP	IgG/IgM Sens: 98.1%, IgG/IgM Spec: 98.6%	CLIA
WANTAI SARS-CoV-2 Ab ELISA	Beijing Wantai Biological Pharmacy Enterprise Co., Ltd	Pan-Ig	S	Pan-Ig Sens 0–7 days: 55.4% 8–14 days: 84.8% ≥ 15 days: 98.7%, Pan-Ig Spec: 97.5%	ELISA
Platelia SARS-CoV-2 Total Ab	Bio-Rad Laboratories, Inc	Pan-Ig	NP	Pan-Ig Sens 0–7 days: 100% 8–14 days: 96% ≥ 15 days: 100%, Pan-Ig Spec: 99.3%	ELISA
SARS-CoV-2 RBD IgG test	Emory Medical Laboratories	IgG	S	IgG Sens 0–7 days: 73% 8–14 days: 100% ≥ 15 days: 100%, IgG Spec: 97.7%	ELISA
SARS-CoV-2 ELISA (IgG)	EUROIMMUN	IgG	S	IgG Sens 0–4 days: 21.7% 5–10 days: 69.4% ≥ 11 days: 81.1%, IgG Spec: 100%	ELISA
cPass SARS-CoV-2 Neutralization Antibody Detection Kit	GenScript USA Inc	Pan-Ig	S	Pan-Ig Sens: 100%, Pan-Ig Spec: 100%	ELISA
SCoV-2 Detect IgG ELISA	InBios International, Inc	IgG	S	IgG Sens 8–14 days: 100% ≥ 15 days: 95.5%, IgG Spec: 100%	ELISA
SCoV-2 Detect IgM ELISA	InBios International, Inc	IgM	S	IgM Sens 0–7 days: 66.7% 8–14 days: 91.4% ≥ 15 days: 93.8%, IgM Spec: 98.8%	ELISA
COVID-SeroKlir, Kantaro Semi-Quantitative SARS-CoV-2 IgG Antibody Kit	Kantaro Biosciences, LLC	IgG	S	IgG Sens 0–7 days: 100% 8–14 days: 100% ≥ 15 days: 93%, IgG Spec: 99.6%	ELISA
Mt. Sinai Laboratory COVID-19 ELISA Antibody Test	Mount Sinai Hospital Clinical Laboratory	IgM, IgG	S	Combined Sens: 92.5%, Combined Spec: 100%	ELISA
Simoa Semi-Quantitative SARS-CoV-2 IgG Antibody Test	Quanterix Corporation	IgG	S	IgG Sens 0–7 days: 45.2% 8–14 days: 87.5% ≥ 15 days: 100%, IgG Spec: 99.2%	ELISA
Dimension Vista SARS-CoV-2 Total Ab assay (COV2T)	Siemens Healthcare Diagnostics	Pan-Ig	S	Pan-Ig Sens 0–6 days: 66.7% 7–13 days: 97.4% ≥ 14 days: 100%, Pan-Ig Spec: 99.8%	ELISA
Dimension EXL SARS-CoV-2 Total Ab assay (CV2T)	Siemens Healthcare Diagnostics	Pan-Ig	S	Pan-Ig Sens 0–6 days: 68.8% 7–13 days: 97.4% ≥ 14 days: 100%, Pan-Ig Spec: 99.9%	ELISA
COVID-19 self-collected Ab test system	Symbiotica, Inc	IgG	S	IgG Sens 8–14 days: 100% ≥ 15 days: 100%, Pan-Ig Spec: 98.04%	ELISA
OmniPATH COVID-19 Total Antibody ELISA Test	Thermo Fisher Scientific	Pan-Ig	S	Pan-Ig Sens 0–7 days: 19% 8–14 days: 76.7% ≥ 15 days: 100%, Pan-Ig Spec: 100%	ELISA
COVID-19 ELISA pan-Ig Antibody Test	University of Arizona Genetics Core for Clinical Services	Pan-Ig	S	Pan-Ig Sens ≥ 15 days: 97.5%, Pan-Ig Spec: 99.1%	ELISA
ZEUS ELISA SARS-CoV-2 Total Test	ZEUS Scientific, Inc	Pan-Ig	S	Pan-Ig Sens: 93.3%, Pan-Ig Spec: 100%	ELISA
ZEUS ELISA SARS-CoV-2 IgG Test System	ZEUS Scientific, Inc	IgG	S	IgG Sens 0–7 days: 100% 8–14 days: 100% ≥ 15 days: 100%, IgG Spec: 99.1%	ELISA
CareStart COVID-19 IgM/IgG	Access Bio, Inc	IgM, IgG	S, NP	IgM Sens 8–14 days: 100% ≥ 15 days: 88.7%, IgM Spec: 99.5% IgG Sens 8–14 days: 100% ≥ 15 days: 96.8%, IgG Spec: 99.5%	LFIA
Assure COVID-19 IgG/IgM Rapid Test Device	Assure Tech	IgM, IgG	S, NP	IgG/IgM Sens 0–7 days: 100% 8–14 days: 83.3% ≥ 15 days: 89.3%, IgG/IgM Spec: 100%	LFIA
ACON SARS-CoV-2 IgG/IgM Rapid Test	ACON Laboratories, Inc	IgM, IgG	S, NP	IgG/IgM Sens 0–7 days: 100% 8–14 days: 100% ≥ 15 days: 100%, IgG/IgM Spec: 95.9%	LFIA
RapCov Rapid COVID-19 Test	ADVAITE, Inc	IgG	NP	IgG Sens ≥ 15 days: 93.3%, IgG Spec: 99.5%	LFIA
WANTAI SARS-CoV-2 Ab Rapid Test	Beijing Wantai Biological Pharmacy Enterprise Co., Ltd	Pan-Ig	S	Pan-Ig Sens: 100%, Pan-Ig Spec: 98.8%	LFIA
Tell Me Fast Novel Coronavirus (COVID-19) IgG/IgM Antibody Test	Biocan Diagnostics Inc	IgM, IgG	S, NP	IgM Sens 8–14 days: 88.9% ≥ 15 days: 85.2%, IgM Spec: 98.7% IgG Sens 8–14 days: 100% ≥ 15 days: 100%, IgG Spec: 96.2%	LFIA
Biohit SARS-CoV-2 IgM/IgG Antibody Test Kit	Biohit Healthcare (Hefei)	IgM, IgG	NP	IgM Sens 0–7 days: 33.3% 8–14 days: 83% ≥ 15 days: 97.7%, IgM Spec: 99.5% IgG Sens 8–14 days: 56.6% ≥ 15 days: 96.2%, IgG Spec: 100%	LFIA
qSARS-CoV-2 IgG/IgM Rapid Test	Cellex, Inc	IgM, IgG	S, NP	Combined Sens: 93.8%, Combined Spec: 96%	LFIA
COvAb SARS-CoV-2 Ab Test	Diabetomics, Inc	Pan-Ig	S	Pan-Ig Sens 0–7 days: 41.6% 8–14 days: 84.2% ≥ 15 days: 97.6%, Pan-Ig Spec: 98.78%	LFIA
RightSign COVID-19 IgG/IgM Rapid Test Cassette	Hangzhou Biotest Biotech	IgM, IgG	S	IgG/IgM Sens 0–7 days: 66.7% 8–14 days: 100% ≥ 15 days: 88.9%, IgG/IgM Spec: 100%	LFIA
LYHER Novel Coronavirus (2019-nCoV) IgM/IgG Antibody Combo	Hangzhou Laihe Biotech	IgM, IgG	S	IgM Sens 0–6 days: 100% 7–14 days: 85.7% ≥ 15 days: 99.3%, IgM Spec: 99.4% IgG Sens 7–14 days: 76.2% ≥ 15 days: 98.5%, IgG Spec: 99.4%	LFIA
COVID-19 IgG/IgM Rapid Test Cassette	Healgen Scientific, LLC	IgM, IgG	S	IgM Sens: 100%, IgM Spec: 100% IgG Sens: 96.7%, IgG Spec: 97.5% Combined Sens: 100%, Combined Spec: 97.5%	LFIA
Innovita 2019-nCoV Ab Test (Colloidal Gold)	Innovita (Tangshan) Biological Technology Co., Ltd	IgM, IgG	S, NP	IgG/IgM Sens 0–7 days: 87.9% 8–14 days: 96.6% ≥ 15 days: 100%, IgG/IgM Spec: 98%	LFIA
SCoV-2 Detect IgG Rapid Test	InBios International, Inc	IgG	S	IgG Sens 0–7 days: 92.9% 8–14 days: 81.8% ≥ 15 days: 100%, IgG Spec: 97.7%	LFIA
Orawell IgM/IgG Rapid Test	Jiangsu Well Biotech	IgM, IgG	S	IgG/IgM Sens 8–14 days: 98.2% ≥ 15 days: 100%, IgG/IgM Spec: 98%	LFIA
Rapid COVID-19 IgM/IgG Combo Test Kit	Megna Health, Inc	IgM, IgG	NP	IgM Sens 0–7 days: 66.7% 8–14 days: 77.1% ≥ 15 days: 90.9%, IgM Spec: 99.6% IgG Sens 0–7 days: 62.3% 8–14 days: 85.7% ≥ 15 days: 90.9%, IgG Spec: 99.3%	LFIA
Nirmidas COVID-19 (SARS-CoV-2) IgM/IgG Antibody Detection Kit	Nirmidas Biotech, Inc	IgM, IgG	S	IgG Sens 0–7 days: 27.8% 8–14 days: 76.5% ≥ 15 days: 100%, IgM Sens 0–7 days: 27.8% 8–14 days: 82.4% ≥ 15 days: 97%, IgM/IgG Spec: 84.8%	LFIA
ADEXUSDx COVID-19 Test	NOWDiagnostics, Inc	Pan-Ig	S	Pan-Ig Sens: 93.3%, Pan-Ig Spec: 100%	LFIA
QIAreach Anti-SARS-CoV-2 Total Test	QIAGEN, GmbH	Pan-Ig	S	Pan-Ig Sens: 100% Pan-Ig Spec: 97.5%	LFIA
Sienna-Clarity COVIBLOCK COVID-19 IgG/IgM Rapid Test Cassette	Salofa Oy	IgM, IgG	S	IgM Sens: 90%, IgM Spec: 100% IgG Sens: 93.3%, IgG Spec: 98.8% Combined Sens: 93.3%, Combined Spec: 98.8%	LFIA
SGTi-flex COVID-19 IgG	Sugentech, Inc	IgG	S, NP	IgG Sens 0–7 days: 41.2% 8–14 days: 91.7% ≥ 15 days: 98.6%, IgG Spec: 100%	LFIA
TBG SARS-CoV-2 IgG/IgM Rapid Test Kit	TBG Biotechnology Corp	IgM, IgG	S, NP	IgM/IgG Sens ≥ 15 days: 96.4%, IgM/IgG Spec: 99.8%	LFIA
BIOTIME SARS-CoV-2 IgG/IgM Rapid Qualitative Test	Xiamen Biotime Biotechnology Co., Ltd	IgM, IgG	S	IgM Sens 0–7 days: 55.1% 8–14 days: 94.1% ≥ 15 days: 100%, IgG Sens 0–7 days: 46.4% 8–14 days: 67.7% ≥ 15 days: 100%, IgM/IgG Spec: 98.5%	LFIA
VIDAS SARS-CoV-2 IgG	BioMérieux SA	IgG	S	IgG Sens 0–7 days: 47.9% 8–14 days: 100% ≥ 15 days: 100%, IgG Spec: 99.9%	ELFA
VIDAS SARS-CoV-2 IgM	BioMérieux SA	IgM	S	IgM Sens 0–7 days: 53.8% 8–14 days: 100% ≥ 15 days: 100%, IgG Spec: 99.4%	ELFA
Maverick SARS-CoV-2 Multi-Antigen Serology Panel v2	Genalyte, Inc	Pan-Ig	S, NP	Ig Sen 0–7 days: 66.7% 8–14 days: 90.9% ≥ 15 days: 96.1%, Ig Spec: 97.7%	PRI
xMAP SARS-CoV-2 Multi-Antigen IgG Assay	Luminex Corporation	IgG	S, NP	IgG Sens 0–7 days: 71.1% 8–14 days: 71.4% ≥ 15 days: 96.2%, IgG Spec: 100%	FMIA
BioPlex 2200 SARS-CoV-2 IgG	Bio-Rad Laboratories	IgG	S	IgG Sens 0–7 days: 81.3% 8–14 days: 96.3% ≥ 15 days: 93.9%, IgG Spec: 99.9%	FIA
FREND COVID-19 total Ab	NanoEntek America, Inc	IgM, IgG	NP	Combined Sens: 96.7%, Combined Spec: 98.8%	FIA
MosaiQ COVID-19 Antibody Magazine	Quotient Suisse SA	Pan-Ig	S	Ig Sens 0–7 days: 100% 8–14 days: 100% ≥ 15 days: 93%, Ig Spec: 99.8%	PIA
Elecsys Anti-SARS-CoV-2	Roche Diagnostics, Inc	Pan-Ig	NP	Ig Sens 0–6 days: 60.2% 7–13 days: 85.3% ≥ 14 days: 99.5%, Ig Spec.: 99.7%	ECLIA
Elecsys Anti-SARS-CoV-2 S	Roche Diagnostics, Inc	Pan-Ig	S	Ig Sens 0–7 days: 90.6% 8–14 days: 87% ≥ 15 days: 96.6%, Ig Spec.: 100%	ECLIA
New York SARS-CoV Microsphere Immunoassay for Antibody	Wadsworth Center, New York State Department of Health	Pan-Ig	NP	Ig Sens 0–6 days: 17.9% 7–10 days: 31.3% 11–15 days: 48.9% 16–20 days: 49.2% > 20 days: 79.3%, Ig Spec: 99.6%	MIA

*CLIA* chemiluminescence immunoassay, *ECLIA* enzyme-enhanced chemiluminescence immunoassay, *ELFA* enzyme-linked fluorescence assay, *FMIA* fluorescent microsphere immunoassay, *FIA* fluorescence immunoassay, *LFIA* lateral flow immunoassay, *MIA*: magnetic immunoassay, *NP* nucleoprotein, *PIA* photometric immunoassay, *PRI* photonic ring immunoassay, *S* spike protein, *Sens* sensitivity (positive percent agreement), *Spec* specificity (negative percent agreement). The commercial kits granted EUA are updated based on the FDA. For each type of method, the products are listed in alphabetical order of the company names

In the next section, we will introduce prominent immunoassays, including ELISAs, CLIAs, and LFIAs, and comprehensively list the applications that have been granted EUA by the U.S. FDA for use as diagnostics for detection of SARS-CoV-2 and serology tests (Tables 4 and 5).

**Table 5:** Antigen lateral flow assays with EUA from U.S. FDA

Test name	Company	LoD (TCID_50_/ml)	Target	Device	SARS-CoV-2 accuracy information	Type of sample
BinaxNOW COVID-19 Ag Card Home Test	Abbott Diagnostics Scarborough, Inc	140.6	NP	NAVICA™ Mobile App	Sens: 91.7% Spec: 100%	ns
BinaxNOW COVID-19 Ag Card	Abbott Diagnostics Scarborough, Inc	140.6	NP	N/A	Sens: 97.1% Spec: 98.5%	ns
CareStart COVID-19 Antigen test	Access Bio, Inc	800	NP	N/A	Sens: 88.4% Spec: 100%	np
NIDS® COVID-19 Antigen Rapid Test Kit	ANP Technologies, Inc	311	NP	N/A	Sens: 95.1% Spec: 97%	ns
BD Veritor System for Rapid Detection of SARS-CoV-2	Becton Dickinson & Company	140.0	NP	BD Veritor Plus Analyzer	Sens: 84% Spec: 100%	ns
BD Veritor™ At-Home COVID-19 Test	Becton Dickinson & Company	187	NP	Scanwell Health App	Sens: 84.6% Spec: 99.8%	ns
CelltrionDiaTrust™ COVID-19 Ag Rapid Test	Celltrion USA, Inc	32	NP, RBD	N/A	Sens: 93.3% Spec: 99.0%	np
Ellume COVID-19 Home Test	Ellume Limited	6309	NP	Ellume COVID-19 Home Test App	Sens: 95% Spec: 97%	ns
GenBody COVID-19 Ag	GenBody Inc	111	NP	N/A	Sens: 91.1% Spec: 100%	np
SCoV-2 Ag Detect Rapid Test	InBios International, Inc	6300	NP	N/A	Sens: 86.7% Spec: 100%	ns
iHealth COVID-19 Antigen Rapid Test	iHealth Labs, Inc	20,000	NP	N/A	Sens: 94.3% Spec: 98.1%	ns
Clip COVID Rapid Antigen Test	Luminostics, Inc	88	NP	Clip COVID Rapid Antigen Test	Sens: 96.9% Spec: 100%	ns
InteliSwab COVID-19 Rapid Test	OraSure Technologies, Inc	250	NP	N/A	Sens: 84% Spec: 98%	ns
InteliSwab COVID-19 Rapid Test Pro	OraSure Technologies, Inc	250	NP	N/A	Sens: 84% Spec: 98%	ns
Status COVID-19/Flu	Princeton BioMeditech Corp	2700	NP	N/A	Sens: 93.9% Spec: 100%	np
INDICAID COVID-19 Rapid Antigen Test	PHASE Scientific International, Ltd	2800	NP	N/A	Sens: 84.4% Spec: 96.3%	ns
QIAreach COVID-19 Rapid Antigen	QIAGEN GmbH	50,000	NP	QIAreach eHub	NP: Sens: 80.7% Spec: 98.3% NS: Sens: 85% Spec: 99.1%	np, ns
Sofia 2 SARS Antigen FIA	Quidel Corporation	113	NP	Sofia 2	Sens: 96.7% Spec: 100%	np, ns
Sofia 2 Flu + SARS Antigen FIA	Quidel Corporation	91.7	NP	Sofia 2	Sens: 95.2% Spec: 100%	np, ns
QuickVue SARS Antigen Test	Quidel Corporation	7570	NP	N/A	Sens: 96.6% Spec: 99.3%	ns
QuickVue At-Home COVID-19 Test	Quidel Corporation	19,100	NP	N/A	Sens: 84.8% Spec: 99.1%	ns
Sienna-Clarity COVID-19 Antigen Rapid Test Cassette	Salofa Oy	1250	NP	N/A	Sens: 87.5% Spec: 98.9%	np

*LoD* limit of detection, *NP* nucleoprotein, *np* nasopharyngeal, *ns* nasal, *Sens* sensitivity (positive percent agreement), *Spec* specificity (negative percent agreement). The commercial kits granted with EUA are updated on the FDA and FIND websites. The assays are listed in alphabetical order of the company names

### Enzyme-linked immunosorbent assay (ELISA)

The four main types of immunoassays include direct, indirect, sandwich, and competitive methods [177]. Most EUAs granted for Ab-based detection tests utilize the indirect ELISA strategy and probe for different human isotype immunoglobulins, such as IgG, IgM, and IgA. For example, some tests to detect virus in human serum or plasma consist of microplates coated with recombinant viral S1 protein. The interaction of antigen and Ab creates an immune-complex, which can be detected using horseradish peroxidase-conjugated Ab and tetramethylbenzidine substrate in a colorimetric reaction [178].

### Chemiluminescence immunoassay (CLIA)

Indirect CLIAs use recombinant antigen-coated magnetic beads as a solid phase, which is incubated with liquid samples containing Ab to create immune-complexes. After the immune-complexes are formed, an enzyme-labelled anti-human Ab is added with the substrate to initiate a chemiluminescence reaction. The result is measured in relative light units and allows for quantification of Abs in the sample. CLIAs are conceptually similar to ELISAs but have a faster average time-to-result, i.e., 1–2 h for CLIA versus 3–5 h for ELISA [179]. In addition, Bastos et al. argued that CLIAs are generally more accurate than traditional ELISAs, according to a systematic review and meta-analysis [180].

### Point-of-care rapid tests (POCTs) using lateral flow immunoassay (LFIA)

POCTs should be easy to operate, portable, and long-lasting, allowing patients to receive test reports in the care setting, rather than days later due to the need of transporting samples to a testing laboratory. POCTs LFIA can be conducted within 10 to 30 min, and it is measured by portable devices or visual observation, making it a potential application for large-scale surveys. However, LFIAs do not have signal amplification, resulting in low signal at low viral or Ab titers. Thus, in comparisons of serology tests, LFIAs often display lower sensitivity than ELISAs and CLIAs [180].

**Fig. 3:**
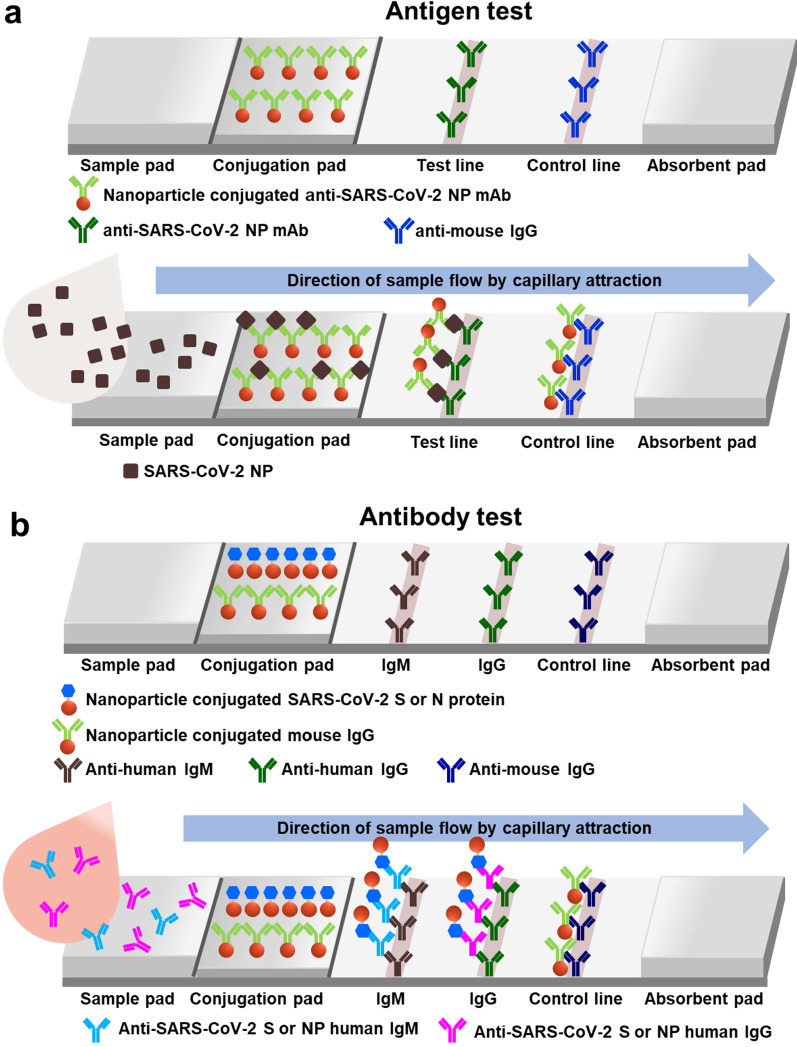
Example of COVID-19 lateral flow immunoassays (LFIA). **a** For antigen detection, a sample containing viral antigens is dropped on the sample pad and flows by capillary action up to the absorbent pad. The sample with viral N protein (NP) directly binds to the anti-NP Ab conjugated with nanoparticles, such as colloidal gold particles or latex nanocomposites. Then, the nanoparticle-conjugated immunocomplexes are released from the conjugation pad. The free epitope of NP is captured to the second anti-NP Ab in the test line. Unbound conjugated Abs will be recognized by immobilized anti-mouse IgG in the control line. **b** For Ab detection, patient serum or plasma specimens are dropped on the sample pad. The sample fluid flows through the conjugation pad which contains nanoparticle-conjugated SARS-CoV-2 spike (S) or N proteins to form antigen-Ab immunocomplexes. The immunocomplexes flow to the test line and are then captured by specific isotype immunoglobulins such as IgM and IgG. Unbound control nanoparticle-conjugated mouse IgG is captured by anti-mouse IgG at the control line

Typical LFIA formats involve capillary migration of sample through immunochromatography paper from a sample pad through a conjugation pad, where conjugated materials are released, and finally migrate to detection lines up to an absorbent pad [181]. An example LFIA with sandwich design for SARS-CoV-2 antigen detection is illustrated in Fig. 3a. NP serves as the target, and the conjugation pad contains capture Abs against NP, which are coupled to colored nanoparticles (e.g., colloidal gold or colored latex) [182]. Another detection Ab against a different epitope of NP is immobilized on the test line, and an anti-mouse IgG Ab is immobilized on the control line. When a sample containing viral NP is loaded, the anti-NP Ab on the conjugation pad captures the antigen, forming an immunocomplex. In this immunocomplex, the free epitope on NP is able to bind the second immobilized detection Ab at the test line. Unbound Abs (not in the immunocomplex) will bind to immobilized Abs on the control line, which captures the Fc of immunoglobulin. Several LFIA-based antigen detection tests for SARS-CoV-2 have been granted EUAs by the U.S. FDA (Table 5). In another example of a serology test (Fig. 3b), recombinant purified SARS-CoV-2 antigen protein (such as S protein) is labeled with nanoparticles and contained in the conjugation pad. Specific Abs from patient serum or plasma specimens are able to conjugate the labeled S protein and then bind to test lines recognizing specific isotype immunoglobulins, such as IgM, IgG, or IgA.

## Spike (S) protein structure-based Abs against SARS-CoV-2

The S protein of SARS-CoV-2 mediates host recognition and membrane fusion, so it has become a major target for the design of drugs and vaccines. S protein is a heavily glycosylated homo-trimeric membrane protein consisting of an extracellular S1 domain, an S2 domain, and a short cytosolic tail. The RBD is located at the top of the S1 domain and can fold as either an open or closed form [183, 184]; in the closed form, the RBM faces the N-terminal portion of its neighboring S1 protomer, while in the open form, it faces up. Consequently, the RBM of an open RBD can bind to the peptidase domain of ACE2, resulting in a trapping of the RBD in its open form and increased S1 subunit conformational dynamics [185]. Notably, the SARS-CoV-2 S trimer is much more sensitive than the SARS-CoV S trimer, with regard to ACE2 receptor-triggered transformation from the closed prefusion state to the fusion-prone open state; this difference might potentially account for the higher infectivity of SARS-CoV-2 compared to SARS-CoV [183, 186].

### Structural biology of S protein

Since the beginning of the pandemic, development of nAbs to block virus entry has included utilization of structural biology tools like crystallography and cryo-EM single particle analysis to reveal viral protein structure and binding epitopes. To investigate the mechanisms of membrane fusion, the structures of various S protein isoforms and conformations have been solved by cryo-EM. Wrapp et al. solved the extracellular domain of S protein by cryo-EM, overcoming low S protein yield by removing the furin cleavage site and introducing two additional proline mutations for stability [63]. Another group reported the cryo-EM structure of full-length S protein, which includes the transmembrane domain and the cytosolic tail [187]. Ke and colleagues used cryo-EM tomography and single particle analysis to reconstruct the S structure directly from deactivated authentic SARS-CoV-2 virus containing the D614G mutation [184]. This structure was then superimposed with the extracellular domain structure. Despite lacking the S transmembrane domain and cytosolic tail, the cryo-EM structure for the extracellular domain showed good agreement with the structure of the S protein from the authentic virus. To gain a better understanding of the molecular conformation of the S protein during the infection process, Xu et al. solved the structure of the extracellular domain of S protein in complex with ACE2 [185]. Comparing the structure of the ACE2-S complex with the closed form of S and the one RBD up open form, the presence of ACE2 induced a swing motion in the ACE2-RBD interface and untwisting of the S trimer; as a result of this increased mobility, a missing density of the neighboring protomer fusion peptide was noted. Such a shift from a packed state to a dynamic state might make the TMPRSS2 cleavage site vulnerable to TMPRSS2 cleavage, thereby initiating the transition to postfusion and host membrane fusion [14].

### Crystal structure of ACE2-RBD complex

The crystal structure of the RBD in complex with ACE2 suggests a molecular mechanism for the initial step of SARS-CoV-2 infection [188]. Between the antiparallel beta-sheet β4 and β7 of the RBD, there is an extended insertion that plays an important role influencing the residues of SARS-CoV-2 S protein that bind to ACE2; these residues are defined as the RBM and include: K417, Y453, Q474, F486, Q498, T500, and N501 (Fig. 1a). The RBDs of SARS-CoV-2 and SARS-CoV share 73% sequence similarity (Fig. 1b), and both viruses bind to ACE2 in an essentially identical manner [189, 190]. However, the RBM of SARS-CoV only shares 49% and 47% sequence similarities with wild-type and Delta SARS-CoV-2, respectively (Fig. 1b). These differences in the RBM sequence markedly increase the ACE2 binding affinity and infectivity of SARS-CoV-2 [53, 191–193]. Additionally, previous studies have shown that nAbs with overlapping epitopes can abolish binding between the RBD and ACE2 [41, 50, 72, 194]. Because the S protein on the SARS-CoV-2 membrane is the key viral component mediating receptor-binding and viral-host cell fusion, nAbs that specifically target the RBD or the RBM have garnered major attention as promising tools to block the fusion between SARS-CoV-2 and host cells.

### Structure of nAb-S/RBD complexes

To summarize the recently published SARS-CoV-2-nAbs (Table 1), we defined three groups based on epitope mapping: Abs that (1) directly bind the RBM, (2) bind the RBD outside the RBM, or (3) bind S protein outside the RBD (Fig. 2). Among the reported structures for Abs in complex with SARS-CoV-2 S protein, most show S protein with 3-RBD down/closed, or 1-RBD up/open and 2-RBD down/closed. Only after stabilizing S protein with mutations do researchers see purified soluble S protein trimers with a 2-RBD up/open or all-RBD up/open conformation that could possibly lead to a more lethal SARS-CoV-2 infection [195]. Additionally, the S protein can display either RBD upward or downward conformations depending on pH. Under physiological conditions (pH 7.4), about 70% of the S protein RBDs have an upward conformation [196]. By lifting the RBD upward, a larger binding surface is made available to nAbs.

Using our classification system, the third group of nAbs may be effectively used in therapeutic combinations with Abs from the first or second groups. However, structural analyses predicting whether nAbs have overlapping epitopes showed that S protein has a dynamic nature, with movement of the NTD, RBD, S2 domain, and the stalk domain in different conformations. Thus, it may be insufficient to only examine Ab-RBD structures or even static images of the S trimer; one must also consider and test for possible simultaneous engagement of nAbs on S proteins with different combinations of up and down RBDs [184, 197]. With these data, researchers may sufficiently understand the SARS-CoV-2 S protein-Ab complexes and proceed to develop novel therapeutic measures against SARS-CoV-2.

### S protein mutations

**Fig. 4:**
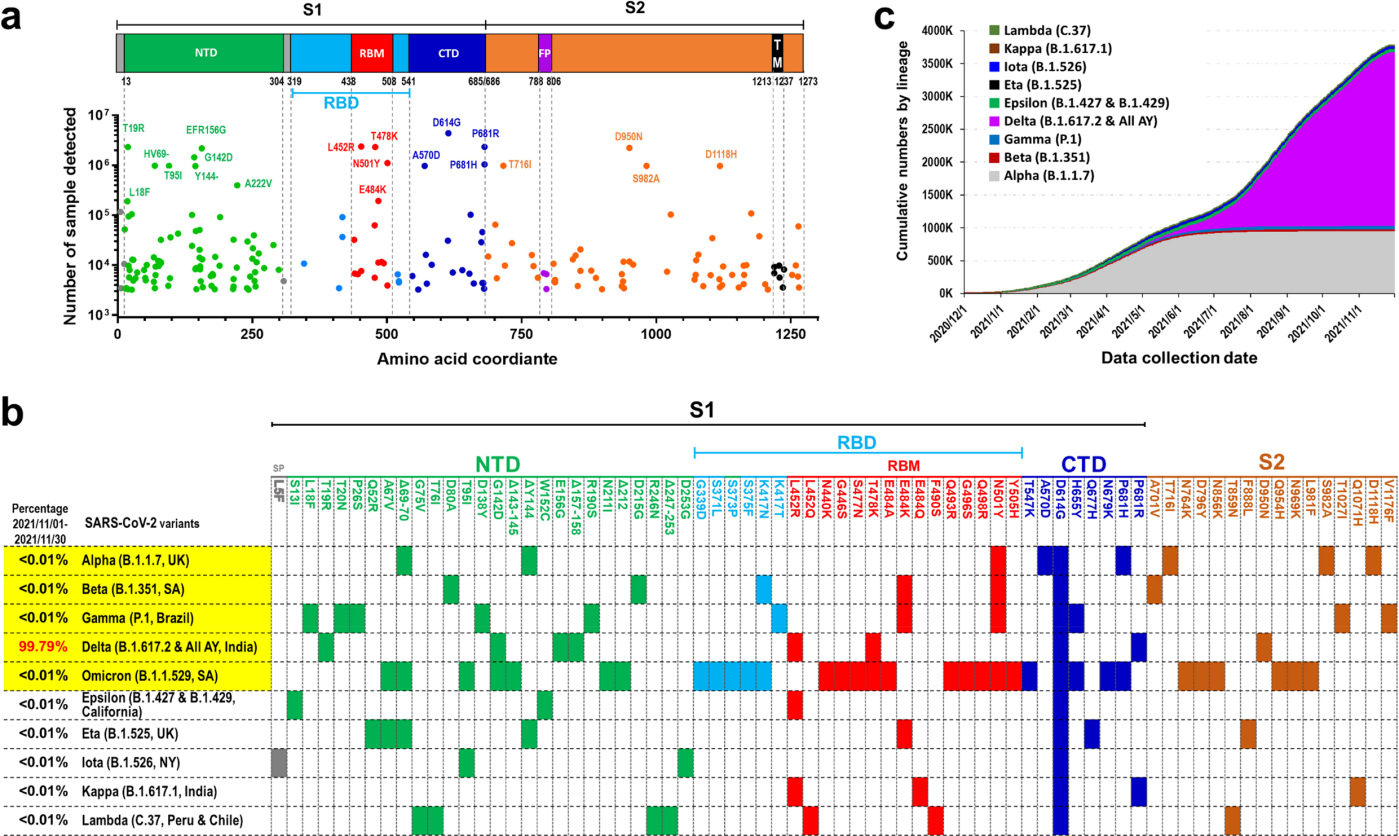
SARS-CoV-2 spike mutations. **a** Top 200 identified SARS-CoV-2 spike mutations. Each dot indicates an amino acid mutation in the S protein. The colors indicate different domains of the SARS-CoV-2 spike protein; *NTD* N-terminal domain, *RBD* receptor-binding domain, *RBM* receptor-binding motif, *CTD* C-terminal domain, *S2* subdomain 2, *FP* fusion peptide, *TM* transmembrane region. The altered amino acids of the top 20 SARS-CoV-2 spike mutations are shown as indicated. The data from 4,450,473 sequences were collected from GISAID and COVID CG (updated to 2021-11-22). **b** Nonsynonymous mutation positions in spike protein of newly emerged SARS-CoV-2 variants. The B.1.1.7 (Alpha), B.1.351 (Beta), P.1 (Gamma), B.1.617.2 (Delta) and B.1.1.529 (Omicron) variants are classified as variants of concern by WHO. The percentage of India B.1.617.2 (Delta) variant includes B.1.617.2 and its all AY sub-lineages. **c** Confirmed COVID-19 cases comprising SARS-CoV-2 variants. The data from 4,337,516 sequences were collected from GISAID and COVID CG (from 2020-12-01 to 2021-11-30) and grouped by lineage

As an RNA virus, SARS-CoV-2 has a higher mutation rate than typical DNA viruses. Up to now, more than 4.45 million viral genomes have been sequenced from COVID-19-positive patients and uploaded to GISAID database (covidcg.org), and hundreds of mutations have been identified in S protein (Fig. 4a). Certain amino acid replacements might change the folding structure or conformation of a protein, potentially leading to increased virulence or evolutionary advantage. Among S protein mutants, those with the D614G point mutation are the most frequently identified in patient samples (Fig. 4a, Table 6), and this mutant has become the one of the dominant mutations of all new emerging SARS-CoV-2 variants worldwide (Fig. 4b). The mutated residue is located within the S1 domain, situated near the fusion peptide of the neighboring protomer (Fig. 1a). Cryo-EM analysis of D614G mutant S protein revealed a looser packing of the trimer structure and a more open form RBD [195]. Compared to the original SARS-CoV-2 S protein, the D614G mutation renders the S protein more stable and reduces the tendency for premature shape change [186]. Although this mutation causes weaker binding between S and the ACE2 receptor, the stability afforded by less frequent premature conformation changes makes the virus more infectious. Another mutation in S protein, A222V, occurred in the dominant D614G strain, and frequently appeared in patients from the recent second wave of the pandemic in Europe [198]. Currently, no structural analysis or patient data suggests that the protein structure of the D614G A222V S protein is different from D614G alone, nor is there evidence that the addition of the A222V mutation further increases infectivity.

**Table 6:** Top 20 identified global SARS-CoV-2 spike protein mutations

Mutant amino acid position	Domain of S protein	Number of sequences detected	Percentage in total cases (%)	Latest month increased (2021/10/22-11/22)
D614G	S1 CTD	4,389,691	96.47	5.0
L452R	RBM	2,370,119	52.09	9.6
P681R	S1 CTD	2,330,791	51.22	9.9
T478K	RBM	2,315,734	50.89	9.9
T19R	S1 NTD	2,311,542	50.80	9.9
D950N	S2	2,230,596	49.02	10.1
EFR156G	S1 NTD	2,178,419	47.87	10.3
G142D	S1 NTD	1,443,479	31.72	13.7
N501Y	RBM	1,103,940	24.26	0.0
P681H	S1 CTD	1,038,019	22.81	0.0
HV69-	S1 NTD	980,548	21.55	0.0
T716I	S2	978,901	21.51	0.0
A570D	S1 CTD	972,330	21.37	0.0
S982A	S2	971,209	21.34	0.0
T95I	S1 NTD	970,839	21.33	0.0
D1118H	S2	970,596	21.33	1.0
Y144-	S1 NTD	960,965	21.12	13.3
A222V	S1 NTD	395,936	8.70	6.5
E484K	RBM	192,337	4.23	0.0
L18F	S1 NTD	190,391	4.18	0.5

*S1* S1 subdomain, *NTD* N-terminal domain, *RBD* receptor-binding domain, *RBM* receptor-binding motif, *CTD* C-terminal domain, *S2* subdomain 2. The data for 4,450,473 sequences from the COVID-19 started to November 22, 2021 were collected from GISAID and COVID CG

At the beginning of 2021, SARS-CoV-2 lineage B.1.1.7 (Alpha) received much attention because it is not only more transmissible than previous variants, but it also leads to increased mortality [199, 200]. Compared to patients with the original virus, B.1.1.7 (Alpha)-infected patients have higher viral loads and show less effective clearance by their immune responses [201]. It was found that this strain contains multiple mutations in the S1 NTD: deletion 69–70, deletion 144; RBM: N501Y; CTD: A570D, D614G, P681H; S2 domain: T716I, S982A, D1118H [202]. As one of the key residues of the RBD that interacts with ACE2 and nAbs (Fig. 4b) [203], mutation of N501 has been shown to increase ACE2 receptor affinity [91]. In particular, tyrosine substitution of asparagine (N501Y) was shown to not only enhance the binding affinity between S protein and ACE2, but it also increases virulence in mice [204–206]. In addition, amino acids 69 and 70 are commonly deleted from the NTD, often in combination with other mutations [207, 208], and the deletions may allosterically change the S protein conformation [63]. These deletions have been found to decrease the viral neutralization by serum from SARS-CoV-2 convalescent patients but not by serum from mRNA-1273 (Moderna)-vaccinated individuals [208, 209]. Another important site is the proline at position 681 (P681), within the furin cleavage site of S protein that exists between the receptor-binding and fusion domains [14]. Although it has not been shown to influence viral entry or transmission, the P681H mutation causes S protein cleavage to occur more efficiently [210].

Additionally, the South Africa lineage (B.1.351, Beta) includes three mutations in the RBD: K417N, E484K, and N501Y (two are in the RBM: E484K and N501Y); one in S1 NTD: D80A; one in S1 CTD: D614G and on in the S2 domain: A701V (Fig. 4b) [211]. This variant became dominant in the South African populations over the course of just a few weeks. Recently, a French research group found that the B.1.351 (Beta) variant has a significant transmission advantage over B.1.1.7 (Alpha) in some European regions [212]. Another variant of concern, the P.1 (Gamma) lineage, arose in Brazil and carries 17 unique amino acid changes, including five mutations in the S1 NTD: L18F, T20N, P26S, D138Y, R190S; three in the RBD: K417N, E484K, and N501Y (E484K and N501Y are in the RBM); one in S1 CTD: D614G; and two in the S2 domain: H655Y, T1027I (Fig. 4b) [211]. Both B.1.351 (Beta) and P.1 (Gamma) variants contain similar mutations in the RBM or RBD of S protein (K417N or K417T, E484K, N501Y), which may cause important conformational changes. The N501Y mutation is the same as the B.1.1.7 (Alpha) variant in terms of enhancing S protein binding to ACE2 and increasing virulence [204–206]. It has been found that the binding and neutralization effects of many SARS-CoV-2-nAbs can be abolished by the K417N and/or E484K mutations on S protein [206]. These effects may be due to structural changes in the receptor-binding site that prevent the interaction with nAbs. However, the binding sites of CR3022 and S309 are distant from K417 and E484, and their neutralizing abilities were unaffected by mutations at these sites [206].

**Fig. 5:**
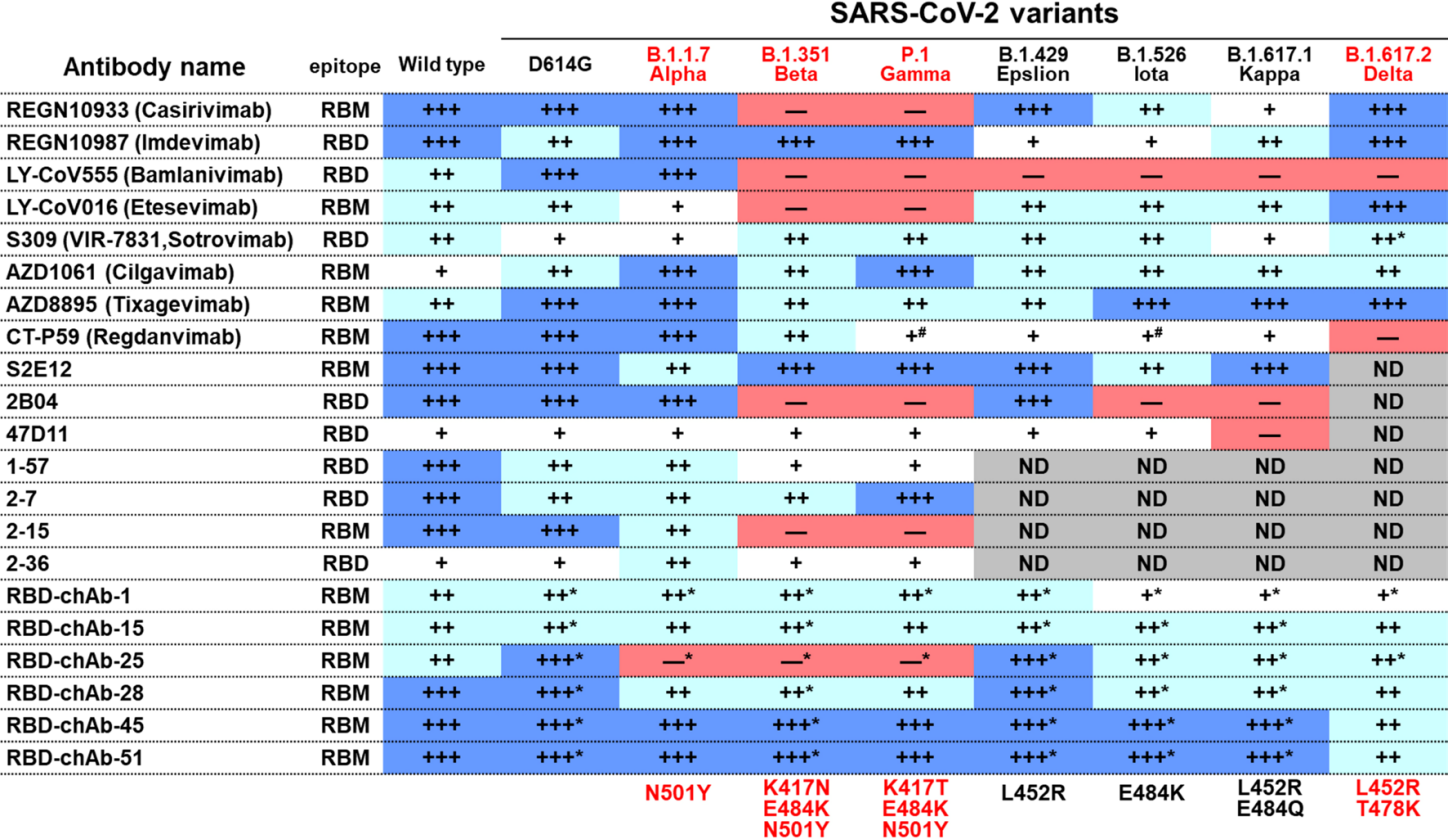
Neutralization of SARS-CoV-2 variants by therapeutic mAbs. The neutralization abilities of therapeutic mAbs against wild-type, D614G and newly emerged SARS-CoV-2 variants, including B.1.1.7 (Alpha), B.1.351 (Beta), P.1 (Gamma), B.1.427 and B.1.429 (Epsilon), B.1.526 (Iota), B.1.617.1 (Kappa), and B.1.617.2 (Delta). Symbols and colors indicate the range of half maximal inhibitory concentration (IC_50_) values toward authentic SARS-CoV-2 virus. +++ with blue, IC_50_ < 10 ng/ml; ++ with light blue, IC_50_ = 10–100 ng/ml; + with white, IC_50_ = 100–1000 ng/ml; —, IC_50_ with red > 1000 ng/ml; ND with grey, no determined; #, preliminary results reported on the website of Celltrion Healthcare Co., Ltd.; *, the range of IC_50_ values toward pseudotype SARS-CoV-2 virus. RBD, receptor-binding domain; RBM, receptor-binding motif. The mutant amino acids in RBD of each SARS-CoV-2 spike protein are shown as indicated

The highly concerning B.1.617.2 (Delta) variant that first emerged in India was shown to be even more transmissible than the B.1.1.7 (Alpha) variant of SARS-CoV-2 (Fig. 4c) [213]. From May to July 2021, this variant spread to many countries at an alarming pace, not only affecting areas with lower vaccination rates, such as southern Africa and Asia, but also causing outbreaks in locations with high vaccination rates, such as the United Kindom and North America [213, 214]. B.1.617.2 (Delta) carries 11 unique amino acid changes in the S protein. The mutations include five in the S1 NTD (T19R, T95I, G142D, deletion 156/157, R158G), two in the RBM of RBD (L452R and T478K), two in the S1 CTD (D614G and P681R) close to the furin cleavage site, and one in the S2 domain (D950N) (Fig. 4b) [29]. It has been shown that B.1.617.2 (Delta) can totally or partially escape neutralization by many antibodies targeting the RBD or NTD of SARS-CoV-2 S protein [215, 216]. Fortunately, some antibodies with EUAs, such as REGN10933 (casirivimab), REGN10987 (imdevimab), and LY-CoV016 (Etesevimab), retain neutralization abilities against the B.1.617.2 (Delta) variant (Fig. 5). Moreover, the newly discovered potent Abs, RBD-chAb-1, 15, 28, 45, and 51, also retain neutralizing ability against the pseudovirus of B.1.1.7 (Alpha), B.1.351 (Beta), P.1 (Gamma), and B.1.617.2 (Delta) lineages of SARS-CoV-2 (Fig. 5) [50]. Therefore, the binding sites of these Abs are promising targets, as they are not subject to interference by SARS-CoV-2 mutations observed to date. Most recently, the B.1.1.529 (Omicron) variant has emerged in South Africa; it is reported to carry a large number of mutations, some of which are concerning [28]. In this variant, there are 32 mutations in the S protein, and 10 of these are within the RBM: N440K, G446S, S477N, T478K, E484A, Q493K, G496S, Q498R, N501Y and Y505H (Fig. 4b) [217]. Based on preliminary evidence that suggests an increased risk of reinfection with this variant, the WHO designated B.1.1.529 as a variant of concern on 26 November 2021 [28]. At the time of this writing, it is still unknown how effective current vaccines and nAbs are at protecting against the Omicron variant, though the topic is under intense investigation.

With regard to nAbs, point mutations or deletions in S protein might result in a local or global conformational change that could potentially disrupt the Ab epitope. The structures of mutated S protein-Ab complexes may reveal global conformational changes caused by the mutation and also whether the nAb can still bind to S protein in the same way as it binds to wild-type S protein. Indeed, the cryo-EM structure of D614G mutant S protein revealed that the RBD is shifted to a more open form as compared to the S protein from the original strain. Such a change in the exposed S protein surface might result in resistance to some Abs. To overcome or postpone the development of drug resistance, some strategies can be considered. The first is to map how all amino-acid mutations in the RBD affect the binding of nAbs [218]. The second approach is to further design escape-resistant Ab cocktails, which would consist of Abs that compete for binding to the same RBD surface but have different escape mutations. Indeed, recent publications have successfully demonstrated synergistic actions of nAbs in vitro. Baum et al. reported a synergistic effect with REGN10987 and REGN10933 in vitro, which was one factor allowing this combination to enter clinical trials even without animal experiments [81]. Despite their use of different neutralization assays, Wu et al. and Pinto et al. both were able to show synergistic effects in vitro for combinations of H4 + B38 and S304 + S309, respectively [72, 97]. Zost et al. further showed a synergistic effect of Abs in a mouse model with adenovirus-induced transient expression of human ACE2 in addition to their in vitro experiments [100]. In addition, Su et al., showed that the cocktail of RBD-chAb-25 and 45 not only exhibits synergistic neutralizing ability, but it is also likely to retain therapeutic potential for SARS-CoV-2 mutants [50, 75]. In addition, some recent studies showed that the accumulation of somatic mutations increased the diversity and potency of neutralizing Abs against SARS-CoV-2 [219–222]. There are also two broadly neutralizing anti-coronavirus antibodies, S2X259 and S2H97, which can neutralize SARS-CoV-2 variants of several pseudoviruses, including B.1.1.7 (Alpha), B.1.351 (Beta), P.1 (Gamma), and B.1.429 (Epsilon) [61, 62]. Therefore, the breadth and potency of nAbs may potentially be improved by using deep mutational scanning to comprehensively identify RBD mutations that lead to escape from binding by each Ab. Such improvements may help to prevent drug-resistant SARS-CoV-2 escape mutants.

## Perspective

As of December 2021, the COVID-19 global pandemic has infected more than 280 million people and caused over 5.40 million deaths [223]. It is expected that the virus will continue to spread and circulate around the world for several years. Serious illnesses and deaths have been reported in all age groups and demographics. However, the percentage of COVID-19 patients that become hospitalized is less than 10% of active cases, except for populations over 65 years of age or with pre-existing diseases [224]. Thus, the majority of individuals with SARS-CoV-2 infection do not require hospitalization or need treatment, and the treatments and policy to control COVID-19 should focus on limiting transmission to reduce the medical burden.

Not only has COVID-19 caused a global health crisis, but it has also profoundly affected the global economy and financial markets [225]. Although more than 8.6 billion doses of SARS-CoV-2 vaccines have been administered around the world, no vaccine can be 100% effective at preventing symptomatic cases of COVID-19; after vaccination, some people may have no or low immune response [226], and others may experience vaccine breakthrough infection [227–229]. In addition, the uneven worldwide distribution of vaccinations, time-limited effects of immunization, and the emergence of new SARS-CoV-2 variants limit the effectiveness of vaccination as a stand-alone strategy to control the pandemic [230]. Even after global mass vaccination has been achieved, some public health measures, such as testing, tracing and isolation of patients still need to be maintained. Otherwise, new waves of infection may lead to even more morbidity and mortality.

Despite the success of COVID-19 vaccination efforts, there is still a need to provide prevention and treatment options for certain populations, including those who cannot be vaccinated or who may have an inadequate response to vaccination. Neutralizing Abs are likely to be critical tools for protecting against SARS-CoV-2 infection, and have been used successfully for this purpose in many animal studies (Table 1); the administration of passive nAbs also has preventive and therapeutic effects for SARS-CoV-2 infection [55, 57, 94]. To prevent work stoppages and alleviate work-related spread of disease, it would be beneficial to have reliable methods to prevent COVID-19 over the course of two to three weeks. Such methods could be used for short-term protection of people who need to work in COVID-19 high-risk areas. These populations can potentially be injected with preventive nAbs in advance of working as usual. There are now four Ab-based treatments authorized for emergency use in adults and teens with mild or moderate symptoms of COVID-19. Among these treatments, bamlanivimab from Eli Lily and REGN-COV2 (casirivimab and imdevimab) were the second (US$871.2 million) and fifth ($185.7 million) best-selling COVID-19 vaccines and drugs of 2020 [231]. In the first half of 2021, bamlanivimab and etesevimab (Eli Lily) and REGN-COV2 were still among the top 10 selling drugs of COVID-19, respectively earning US$959.1 million and $4.156 billion [88]. Analysts have forecasted full-year sales of about $7.0 billion for REGN-COV2 in 2021 [88, 232]. These sales figures suggest that more and more people and doctors believe nAbs have benefits in the prevention and treatment of COVID-19.

As the key determinant of host membrane fusion, S protein has become a major subject of research on SARS-CoV-2 infection mechanisms and a prime target for therapeutic Ab development. As one of only two exposed membrane proteins on SARS-CoV-2, it is also a predominant immunogen for the human immune system. A large scale survey of COVID-19 patient serum samples revealed that 96% to 98% of patient Abs recognize S protein [233], and 76% recognize the RBD in particular [234]. However, RNA viruses exhibit high mutation frequencies in the human body, and an increasing list of SARS-CoV-2 variants have been detected. To date, there have been hundreds of mutations identified in S protein (Fig. 4), and many are rapidly spreading in the population. Some of these point mutations might trigger local or global protein structure changes that enhance virulence or cause loss of efficacy for vaccines and nAbs [27, 53, 192, 235]. Therefore, Ab combination/cocktail therapies should be considered as a strategy to prevent the emergence of SARS-CoV-2 escape mutants. In addition to the REGN-COV2 cocktail Abs (casirivimab and imdevimab) [81], the combination treatment of bamlanivimab with etesevimab also reduced SARS-CoV-2 log viral load at day 11 in patients with mild to moderate COVID-19 [94]. Although it has been reported that casirivimab, bamlanivimab, and etesevimab lose neutralizing activity against B 1.351 (Alpha), and P.1 (Gamma) variants of SARS-CoV-2 [53, 54], the cocktails of Abs with non-overlapping epitopes on the RBD have been shown to exhibit great efficacy for neutralizing SARS-CoV-2 mutant escape variants [50, 76, 85, 236]. Continual development of potent nAbs against new variants of SARS-CoV-2 is therefore urgent and essential. Although no signs of ADE have been reported in human clinical trials, Liu et al. recently reported some specific Abs binding to the NTD of the open RBD might enhance virus infectivity independent of the Fc-receptor [36]. This observation is noteworthy because it means that infected or vaccinated people who generate such Abs may have a higher risk of future virus infection. Therefore, cocktails of Abs targeting multiple non-overlapping and avoiding infectivity-enhancing epitopes are a promising avenue for the development of COVID-19 therapies.

To date, enormous amounts of resources have been dedicated to studies focused on understanding the SARS-CoV-2 S protein and its RBD, to develop new tools to fight COVID-19. Many potent neutralizing mAbs have been shown to effectively inhibit virus binding to the host receptor, hACE2, both in vitro and in vivo. Cocktails of these neutralizing mAbs directed against non-competing epitopes are likely to improve the efficacy of Ab-based treatments while also preventing the emergence of SARS-CoV-2 escape mutants. Along with the ongoing vaccination efforts, easy and cheap detection systems are urgently needed to control disease spread, especially to control the unexpectedly rapid spread of escaped mutant viruses [237]. Antigen and Ab detection tests are promising candidates to fill this need and are widely used in many countries, giving the products an enormous market value [238]. The use of these products can represent a major clinical cost-saving practice, due to their low pricing, convenient and timely detection of infectious diseases with limited or no symptoms, acceleration of decisions regarding treatment or isolation, and reduction of other complications [239]. However, there has never been such a large-scale demand for these types of products, so the production and supply logistics will be key problems that need to be resolved. Assuredly, international partnerships for manufacturing and distribution, as well as new manufacturing platforms, will be required to address this pressing global need.

## Conclusions

The COVID-19 pandemic is an ongoing global disaster and one of the leading causes of death in the past year, a distinction that is unprecedented in recent human history [240]. Fortunately, collaborative efforts within the worldwide scientific community have allowed the extraordinarily rapid development and authorization of vaccines and nAbs against COVID‑19. These successful efforts have greatly benefited from tremendous financial support by the governments of developed countries and the solid R&D and manufacturing capacities of pharmaceutical companies [241–244]. For example, the U.S. government has pre-ordered 100 million doses of Pfizer mRNA vaccine, BNT162b2, at a price of US$1.95 billion as early on July 22, 2020, and after one year, it has already ordered a total of 500 million doses of Pfizer mRNA vaccine. Furthermore, the U.S. government also purchased 100 million doses of mRNA-1273 of Moderna mRNA vaccine for US$2.48 billion as early on August 11, 2020, and up to July 2021, it reached to a total of 500 million doses of mRNA-1273 ordered by the U.S. government. As of February 2021, Sanofi with GlaxoSmithKline and Novavax had together received about US$2.1 billion from public and non-profit funding sources for vaccine development. Around the world, funding agencies have already paid over US$10 billion to vaccine developers [244]. In addition, the developers of therapeutic Abs, such as Regeneron, Eli Lilly and AstraZeneca, have received more than US$100 million for the production of therapeutic Abs against COVID-19 [90, 107, 245]. These extraordinary investments are one of the main reasons that hope for an end to the pandemic is beginning to shine in many countries around the world. However, the widespread emergence of highly communicable variants, such as B.1.617.2 (Delta) and B.1.1.529 (Omicron), and some uncontrolled outbreaks mean that much work remains to be done toward developing effective next-generation vaccines and medicines including therapeutic Abs for COVID-19. The advance purchase of therapeutic Abs by governments will speed the progress of pharmaceutical companies in obtaining authorizations or licensure from the FDA. Then, the clinical deployment of these therapeutic Abs can provide crucial tools for combatting SARS-CoV-2. As the emergence of variant lineages is now one of the most difficult obstacles to controlling the COVID-19 pandemic, the next generation of vaccines and therapeutic Abs must target epitopes on variants. It is especially important to predict and target epitopes with high potential to alter the transmission or infectivity of the virus, including those in recent or future emergent variants of SARS-CoV-2.

## Data Availability

All the data and materials supporting the conclusions were included in the main paper.

## Abbreviations

Ab: Antibody
ACE2: Angiotensin converting enzyme II
ADE: Ab-dependent enhancement
ARDS: Acute respiratory distress syndrome
BLAZE-1: Blocking Viral Attachment and Cell Entry with SARS-CoV-2 Neutralizing Antibodies
CDC: Centers for Disease Control and Prevention
CLIA: Chemiluminescence immunoassay
COVID-19: Coronavirus disease 2019
CPE: Cytopathic effect
CRS: Cytokine release syndrome
CSS: Cytokine storm syndrome
cryo-EM: Cryogenic electron microscopy
ELISA: Enzyme-linked immunosorbent assay
EUA: Emergency use authorization
Fab: Antigen-binding fragment
Fc: Fragment crystallizable
FDA: Food and Drug Administration
FP: Fusion peptide
FPPR: Fusion peptide proximal region
FRNT: Focus reduction neutralization assay
GM-CSF: Granulocyte-macrophage colony-stimulating factor
GM-CSFR-α: GM-CSF receptor-α
IFA: Immunofluorescence assay
IFP: Internal fusion peptide
IgG: Immunoglobulin G
IgM: Immunoglobulin M
IL-1β: Interleukin 1 beta
IL-6: Interleukin 6
IL-6R: IL-6 receptor
LFIA: Lateral flow immunoassay
MAC: Membrane attack complex
mIL-6R: Membrane-bound IL-6 receptor
mAb: Monoclonal antibody
NP: Nucleoprotein
NTD: N-terminal domain
POCT: Point-of-care testing
PRNT: Plaque reduction neutralization test
qPCR: Quantitative polymerase change reaction
RBD: Receptor-binding domain
RBM: Receptor-binding motif
RT-PCR: Reverse transcriptase-polymerase chain reaction
S: Spike
SARS-CoV-2: Severe acute respiratory syndrome coronavirus 2
scFv: Single-chain fragment variable
sIL-6R: Soluble form of IL-6 receptor
TMPRSS2: Transmembrane serine protease 2
TNF: Tumor necrosis factor
